# The transcriptional coactivator Eya1 exerts transcriptional repressive activity by interacting with REST corepressors and REST-binding sequences to maintain nephron progenitor identity

**DOI:** 10.1093/nar/gkac760

**Published:** 2022-09-21

**Authors:** Jun Li, Chunming Cheng, Jinshu Xu, Ting Zhang, Bengu Tokat, Georgia Dolios, Aarthi Ramakrishnan, Li Shen, Rong Wang, Pin-Xian Xu

**Affiliations:** Department of Genetics and Genomic Sciences, New York, NY 10029, USA; Department of Genetics and Genomic Sciences, New York, NY 10029, USA; Department of Genetics and Genomic Sciences, New York, NY 10029, USA; Department of Genetics and Genomic Sciences, New York, NY 10029, USA; Department of Genetics and Genomic Sciences, New York, NY 10029, USA; Department of Genetics and Genomic Sciences, New York, NY 10029, USA; Department of Neurosciences, New York, NY 10029, USA; Department of Neurosciences, New York, NY 10029, USA; Department of Genetics and Genomic Sciences, New York, NY 10029, USA; Department of Genetics and Genomic Sciences, New York, NY 10029, USA; Department of Cell, Developmental and Regenerative Biology, Icahn School of Medicine at Mount Sinai, New York, NY 10029, USA

## Abstract

Eya1 is critical for establishing and maintaining nephron progenitor cells (NPCs). It belongs to a family of proteins called phosphatase-transcriptional activators but without intrinsic DNA-binding activity. However, the spectrum of the Eya1-centered networks is underexplored. Here, we combined transcriptomic, genomic and proteomic approaches to characterize gene regulation by Eya1 in the NPCs. We identified Eya1 target genes, associated *cis*-regulatory elements and partner proteins. Eya1 preferentially occupies promoter sequences and interacts with general transcription factors (TFs), RNA polymerases, different types of TFs, chromatin-remodeling factors with ATPase or helicase activity, and DNA replication/repair proteins. Intriguingly, we identified REST-binding motifs in 76% of Eya1-occupied sites without H3K27ac-deposition, which were present in many Eya1 target genes upregulated in *Eya1*-deficient NPCs. Eya1 copurified REST-interacting chromatin-remodeling factors, histone deacetylase/lysine demethylase, and corepressors. Coimmunoprecipitation validated physical interaction between Eya1 and Rest/Hdac1/Cdyl/Hltf in the kidneys. Collectively, our results suggest that through interactions with chromatin-remodeling factors and specialized DNA-binding proteins, Eya1 may modify chromatin structure to facilitate the assembly of regulatory complexes that regulate transcription positively or negatively. These findings provide a mechanistic basis for how Eya1 exerts its activity by forming unique multiprotein complexes in various biological processes to maintain the cellular state of NPCs.

## INTRODUCTION

Proper spatiotemporal regulation of gene expression plays a central role in developmental processes and cellular function. It is achieved through the activity of transcription factors (TFs) that bind to *cis*-regulatory DNA elements (CREs), which recruit enzymatic chromatin regulatory complexes that condition the chromatin environment in favor of either transcriptional activation or repression. Mutations in TFs, CREs or enzymatic chromatin regulatory complexes that mediate the activity of TFs are associated with a variety of diseases, including cancer and developmental disorders. The TF Eya1 acts at a top of a regulatory network that establishes the metanephric mesenchyme (MM) (1–3) and maintains the MM-derived nephron progenitor cell (NPC) population throughout kidney development (4). Eya1 belongs to the Eyes absent (Eya) protein family that possesses intrinsic tyrosine and threonine/serine phosphatase activities and transcriptional activation functions but without intrinsic DNA-binding ability, thus regulating gene expression through interactions with DNA-binding partner proteins (5,6).

The Eya protein family consists of a conserved C-terminal Eya domain (ED) participating in protein-protein interactions and a highly divergent N-terminus (NT) as a transcriptional activation domain (7,8). In *Drosophila*, Eya interacts with Sine oculis (So) and Dachshund (Dach) to synergistically induce ectopic eye formation (9,10). During mammalian development, the homeodomain Six/So family proteins are the best characterized Eya-interacting proteins (5,11). In the MM progenitors, we previously showed that Eya1 interacts with Six1/Six2 and Myc (4,12,13). Both Six1 and Six2 mediate the nuclear translocation of Eya1 when coexpressed in cultured cells (13) or in the MM progenitors (4,14). Haploinsufficiency for *EYA1* or *SIX1* causes branchio-oto-renal syndrome (15,16), while mutations in *SIX2* result in renal hypodysplasia (17). Recently, we reported that Eya1 and Six2 interact with the ATP-dependent SWI/SNF chromatin remodeling complex to target CREs of NPC-specific genes such as the *Pbx1* and *Myc* to regulate their expression in the NPCs (18). Deletion of either *Eya1*, *Six2* or the ATPase subunit of the SWI/SNF complex *Brg1* causes NPC depletion due to premature differentiation and abnormal cell death (4,18–20). Eya1 and Six family genes are also implicated in cell proliferation as reduced proliferation was observed in the nephrogenic mesenchyme as well as in several other organs associated with *Eya1*- or *Six1;Six4*-deficiency (2,21,22). However, how these genes act to mediate cell proliferation and maintain the NPC identity during kidney development is poorly understood.

EYA’s phosphatase activity has been implicated in proliferation, DNA damage repair, cell migration and cancer metastasis in tumorigenesis (6). Studies have shown that EYA dephosphorylates the DNA damage sensing histone H2A variant H2AX (23,24) and the tumor suppressor estrogen receptor ERβ (25). Eya1’s phosphatase activity was reported to dephosphorylate the proto-oncogene Myc at Thr58 in MM progenitors (4,26) and the Notch intracellular domain at Thr2122 (27) and to promote Shh signaling during development (28). However, it remains unclear how Eya1 regulates gene expression programs to establish and maintain the cellular state of the NPCs and whether Eya1 also interacts with chromatin remodeling complexes to repress or silence expression of disease-associated genes or genes for non-NPC cell types.

In this study, we combined transcriptomic, genomic, and proteomic approaches to characterize three aspects of Eya1: regulated genes, associated CREs and interacting proteins. We identified putative cofactors and targeted CREs and genes for Eya1, many of which have roles in NPC maintenance. We found that Eya1 physically interacts with different classes of chromatin remodeling complexes with ATPase or helicase activity. Eya1 occupies preferentially to promoter sequences and interacts with general transcription factors (TFs), RNA polymerases, different types of TFs, and DNA replication/repair proteins. Intriguingly, we identified REST-binding motifs in 76% of Eya1-occupied sites without H3K27ac-deposition, which were present in many Eya1 target genes upregulated in *Eya1*-deficient NPCs. Eya1 physically interacts with REST and REST-interacting proteins, including histone deacetylase/lysine demethylase and Cdyl. These results provide the first evidence that Eya1 acts as a REST corepressor in the NPCs. Together, our findings provide a mechanistic basis for how Eya1 exerts its activity by forming distinct multiprotein complexes during various fundamental biological processes to maintain the cellular state of NPCs.

## MATERIALS AND METHODS

### Animals

The *Eya1^CreER^* (4), *Eya1^HA-FLAG^* knockin (18) and *R26-tdTomato* mice were maintained on a mixed background of C57BL/6J and 129/Sv. All animal experiments were approved by the Animal Care and Use Committee of Icahn School of Medicine at Mount Sinai (#06-0822). Mice were bred using timed mating and noon on the day of vaginal plug detection was considered as E0.5. For induction of the CreER protein, tamoxifen (T5648, Sigma) was dissolved in corn oil (C8267, Sigma) and administrated (2 mg/10 g body weight) by oral gavage.

### Kidney single cell isolation and fluorescence-activated cell sorting

Kidneys were isolated from E12.5–E12.75 control (*Eya1^CreER^;tdTomato*) or *Eya1*cKO (*Eya1^CreER^;Eya1^fl/^^fl^;tdTomato*) embryos, minced, and digested with dispase (1 mg/ml) and collagenase IV (0.7 mg/ml) in PBS for 20 min at 37°C. After removing the cell clumps by passing through a 40 μm strainer (BD Biosciences), the cell suspension was centrifuged, washed twice in PBS to remove fragments, then resuspended in a freezing medium, and stored in a liquid N2 tank. Twelve kidneys from six embryos were collected from five different pregnant females. After genotyping, we combined cell suspensions for FACS to isolate tdTomato^+^ cells.

### RNA-seq

The total RNAs were prepared from 5000 cells using a NucleoSpin RNA XS kit (Cat.740902, TakaraBio), and then mRNA was purified from total RNA using magnetic beads (S1550S, New England Biolabs). Random priming strand-specific RNA-seq libraries were prepared using the SMARTer stranded RNA-seq kit (Cat.634839, TakaraBio) and sequenced using Illumina NextSeq 500 with single-end 75-bp. The reads were mapped to mouse genome mm10 using HISAT2 (29). HTSeq-count against Ensembl v90 annotation (30) was used for reads counting. Differential expression study was performed using the DESeq2 package (31). Volcano plotting of global expression changes between control and *Eya1*cKO cells was carried out using web tools in Galaxy (https://usegalaxy.org). Gene ontology and pathway analyses were performed using webtool geneontology.org. Heatmap, *K*means, and Kyoto Encyclopedia of Genes and Genomes analyses were performed using http://bioinformatics.sdstate.edu/idep/ (32).

### Reverse transcription (RT) and quantitative real-time PCR (qPCR)

The total RNAs were prepared from 5000 FACS-purified tdToamto^+^ cells of *Eya1^CreER/+^* control or *Eya1^cKO/^^cKO^* kidneys using a NucleoSpin RNA XS kit (Cat.740902, TakaraBio). ∼100–150 ng of total RNAs were used for reverse transcription with a SuperScript IV Reverse Transcriptase (18090010, Thermo Fisher Scientific) for first-Strand cDNA Synthesis. qPCR was performed using SYBR Green Master Mix (4309155, Applied Biosystems). StepOnePlus Real-Time PCR Systems. Expression levels of each transcript were normalized using β-actin as an internal control. Each set of experiments was repeated three times, and the DDCT relative quantification method was used to evaluate quantitative variation. All PCR primers are listed in Supplementary Table S5.

### ChIP-seq

For Eya1 ChIP-seq, 80 kidneys from E13.5 *Eya1^HA-FLAG^* knockin mice were used for each ChIP assay. The kidneys were crosslinked, homogenized, and lysed, and chromatin was sonicated as described (18). Sonicated chromatin was cleared by pelleting insoluble material at 13 000 rpm at 4°C, followed by preclear with protein A/G beads and incubation with ∼20 μl blocked anti-FLAG M2 beads and IgG as a negative control. After washing, FLAG peptide was used for elution. The pull-down and input control sequencing libraries were generated using the ThruPLEX DNA-seq Kit (R400429; Rubicon Genomics) and sequenced on Illumina NextSeq500.

### Peak calling and annotation

Quality controls using FastQC (v0.11.2) (http://www.bioinformatics.babraham.ac.uk/projects/fastqc) were generated, and raw sequencing reads were then aligned to the mouse mm10 genome using default settings of Bowtie (v2.2.0). Peak calling was performed using MACS (v2.1.1) (33) with various *P* value cutoffs. The peak bed files were generated from peak calling against genomic input control or IgG control. The common peaks from these two bed files were used for subsequent analyses. The overlapping peaks of bed files were identified using the bedtools from Galaxy(https://usegalaxy.org/). Motif enrichment analysis was performed using the Homer package (v4.8.3) (34). The peak annotation and GO analysis was performed using the GREAT program (35) and the Panther classification system. The bamCoverage, computeMatrix, and plotHeatmap tools from Galaxy platform were used for a general comparison of overall peaks/signal via the normalized coverage comparisons.

### Generation of 2 × HA-3× FLAG-Eya1 (B23) stable cell line

This cell line was developed by cotransfecting HEK293 cells with 2× HA-3× FLAG-Eya1/pcDNA3 and pBABE. Stable transfectants were selected for 4 weeks in the presence of 3 μg/ml puromycin. Surviving clones were analyzed by western blotting to select HA-FLAG-Eya1-expressing clones.

### Tandem affinity purification and identification of interacting proteins of Eya1

Ten 15-cm diameter dishes of HA-FLAG-Eya1/HEK293 stable cells were washed with PBS, lysed in IP lysis buffer (50 mM Tris–HCl, pH 7.5, 150 mM NaCl, 0.1% Nonidet P-40, 5 mM EDTA, 5 mM EGTA, 15 mM MgCl_2_, 60 mM β-glycerophosphate, 0.1 mM sodium orthovanadate, 0.1 mM NaF, 0.1 mM benzamide, phosphatase and protease inhibitor cocktail from Roche) and rotated for 1.5 h at 4°C. After the cell lysates were centrifuged for 10 min at 12 000 rpm, 4°C, supernatant were precleared with Protein G agarose beads (100 μl of 50% slurry per 10 mg protein) for 1–2 h at 4°C with continuous mixing. Samples were centrifuged for 5 min at 300 × g, 4°C, and the precleared supernatant was transferred to new 50 ml conical tubes containing preequilibrated FLAG M2 beads (M8823, Sigma) and rotated at 4°C overnight, which then subjected to spin to remove supernatant. The FLAG-agarose beads were washed four times with 10 ml ice-cold IP buffer each for 30 min at 4°C. IP buffer was used to transfer beads to a 15 ml tube. Beads were pooled down by centrifuging for 4 min at 300 × g and 4°C and eluted four times, each with 200 μl of IP buffer with 0.1 mg/ml FLAG peptide and rotating for 1–1.5 h at 4°C and centrifuging 4 min at 300 × g and 4°C. The eluted protein supernatant was transferred to a new 1.5 ml tube with added equilibrated anti-HA-agarose beads (88836, Pierce). After overnight rotating at 4°C, HA-beads were span for 4 min at 300 × g and 4°C and washed four times with 1 ml of ice-cold IP buffer, each for 30 min at 4°C. 50 μl of 2 × SDS loading sample buffer was added, and proteins were separated using NuPAGE Bis–Tris Mini Gels at 200 V for 35 min.

Gel was silver stained for Mass Spectrometry (Pierce Silver Stain for Mass Spectrometry: 24600) and subjected to excising to destaining procedure. After washing, the gel pieces were then proceeded with in-gel trypsin digestion or other protein elution steps in preparation for the desired mass spectrometry method.

### Immunohistochemistry (IHC)

IHC on kidney sections was performed according to standard procedures or as described previously (4). Cy3-, Cy5- and FITC-conjugated secondary antibodies were used for detection. Hoechst 3342 was used for nuclear staining.

### CoIP analysis

Cell lysate were prepared from kidneys of E14.5 *Eya1^HA-FLAG^* embryos using lysis buffer (20 mM Tris–HCl, pH 8.0, 150 mM NaCl, 1% NP-40, 10% glycerol, 2 mM EDTA and protease inhibitor) as described previously (36). The lysate was diluted with IP buffer (16.7 mM Tris–HCl, pH 7.3, 50 mM KCl, 1.25 mM MgCl_2_, 0.17 mM EDTA, 20% glycerol and protease inhibitor) to reduce NP-40 concentration to 0.5%. After preclear with protein A/G bead, the lysate was incubated with anti-FLAG M2 beads (F7424, Sigma) overnight. The immunocomplexes were washed 4 times with IP buffer with 0.1% detergent and then separated by SDS-PAGE gel for western blot detection with primary antibodies and HRP-conjugated secondary using the enhanced chemiluminescence (ECL) method (WBKLS0500, Millipore).

### Primary antibodies

Anti-Flag (205431AP, Proteintech: dilution 1:3000 for western), -Six2 (66347, Proteintech: dilution 1:150 for IHC), -Rest (ABIN747683, Antibodies-online: dilution 1:100 for IHC and 1:2000 for western), -Cdyl (17763–1-AP, ThermoFisher: dilution 1:200 for IHC and 1:1000 for western), and -HDAC1 (ab53091, Abcam: dilution 1:2000 for western), and -Hltf (MBP183256, Novus Biologicals: dilution 1:100 for IHC and 1:2000 for western; MABE1074, Millipore-Sigma: dilution 1:60 for IHC).

## RESULTS

### Transcriptome profiling of *Eya1*-lineage cells of control and *Eya1*cKO kidneys reveals *Eya1*-dependend expression of genes essential for NPC maintenance

**Figure 1.**
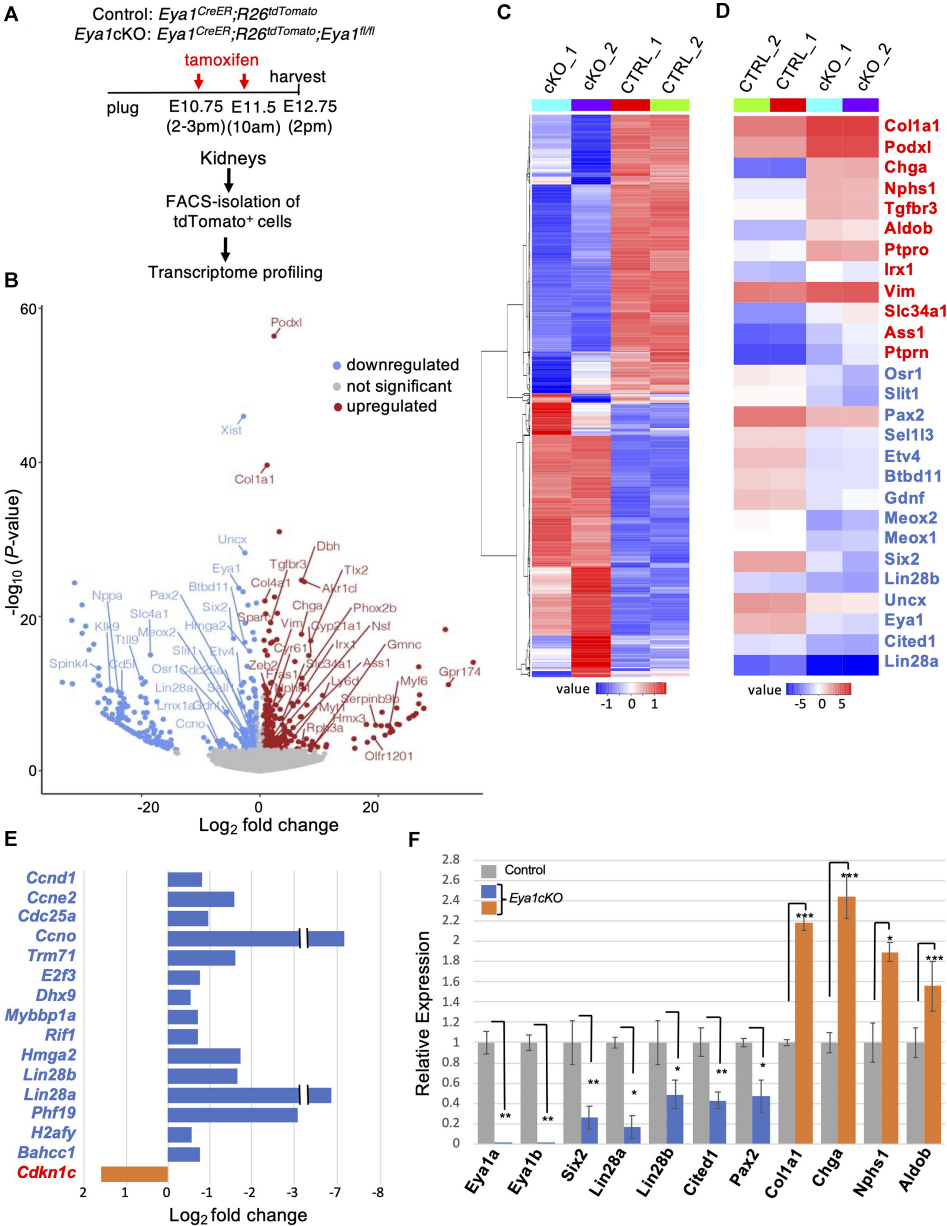
Transcriptome profiling analyses reveal the requirement of Eya1 for NPC maintenance. (**A**) Schematic drawing of tdTomato + cell isolation. Kidneys (*n*= 12) were isolated from control and *Eya1^cKO/^^cKO^* littermate embryos at E12.5–12.75 (*n*= 6 for each genotype harvested from 4 different pregnant females) 48 hrs after initiating tamoxifen administration and dissociated into single cells. Five thousand FACS-sorted tdTomato^+^ cells were used for each biological replicate (samples ‘1’ and ‘2’). (**B**) Volcano plot showing transcripts differentially responding to depletion of Eya1. Genes with *P* adjusted > 0.05 and log_2_ fold change <0.58 are displayed in gray. (**C** and **D**) Heatmap showing expression of all 1181 (C) or 27 selected (D) differentially expressed genes in each sample. Blue and red indicate down- and upregulated genes, respectively, in *Eya1^cKO/^^cKO^*. (**E**) Graph showing log_2_ fold change in selected down- and upregulated differentially expressed genes involved in cell cycle. (**F**) Quantitative RT-PCR using FACS-purified tdTomato cells of *Eya1^CreER/+^* (control) and *Eya1^cKO/^^cKO^* (cKO) kidneys at E12.5–12.75. qPCR was performed in triplicate and repeated three times. *P< 0.05; **P< 0.01; ***P< 0.001.

To identify the downstream genes regulated by Eya1 in NPCs, we performed RNA-seq experiments using FACS-sorted tdTomato^+^ cells from control (*Eya1^CreER/+^;tdTomato*) and *Eya1*cKO (*Eya1^CreER/+^;Eya1^fl/fl^;R26-Tdtomato*) kidneys at ∼E12.75 respectively (harvested ∼48 hrs after tamoxifen treatment) (Figure 1A). Differential expression analysis of *Eya1*cKO versus control identified 1181 differentially expressed genes (DEGs) (619 with adjusted *P* < 0.05 and 562 with log_2_Fold-Change > 1.2 and raw *P* < 0.03) with 520 downregulated and 661 upregulated in *Eya1*cKO (Figure 1B, C, Supplementary Table S1). We noticed that some *Eya1* transcripts were detected by the RNA-seq (Figure 1D), but they were extremely low by qRT-PCR (Figure 1F). This suggests that two doses of tamoxifen administration did not remove 100% of *Eya1* within 48 h, possibly due to the failure of CreER to be efficiently activated simultaneously in all cells, and this residual expression can be detected by deep sequencing due to its high sensitivity. Nonetheless, consistent with previous findings that *Eya1-*deficiency leads to NPC depletion and premature differentiation (4), we observed downregulation of genes necessary for NPC maintenance and upregulation of nephron differentiation genes (Figure 1D). The downregulated genes include *Six2*, *Gdnf, Evt4/Evt5* (target of the Gdnf-Ret pathway)*, Sall1, Hoxa11/Hoxd11, Pax2, Cited1/Cited2, Btbd11* (induced by Wnt9b), *Uncx, Meox1/Meox2*, *Fgfr1, Slit1, Osr1, Sox9/Sox11*, *Hmga2* and the RNA-binding proteins *Lin28a*/*Lin28b* that regulate the timing of cessation of nephrogenesis (37). In contrast, upregulated genes include podocyte lineage-specific genes such as the TF *Efnb* and *Mafb/Magi2/Nphsh1/Nphs2/Ptprn/Ptpro/Podxl/Synpo/Thsd7a/Zbtb7c/Clic5/Rph3a/Tgfbr3* and nephron tubule-specific genes such as *Ass1/Aldob*/*Slc34a1*/*Irx1/Zeb2/Serpinb9b* and *Chga/Chgb* (elevated in patients with kidney dysfunction). Upregulation of genes associated with other diseases, renal dysfunction or cancer was also observed, such as *Fras1* (Fraser syndrome 1), *Cyp21a1* (congenital adrenal hyperplasia), *Dbh* (associated with kidney dysfunction), and *Akr1c1/Myt1/Myf6/Scg2* (kidney cancer) (Figure 1B). Furthermore, numerous TFs involved in neurogenesis or cardiac development such as *Otx2*, *Phox2a/Phox2b*/*Tlx2*, *Sox2/Sox10/Sox17, Isl1/Isl2, Tbx5/Tbx2* and *Hand1/Hand2* were also upregulated. Additionally, increased expression of genes related to extracellular matrix (ECM) and fibrosis, such as collagens *Col1a1/Col4a1*, *Vim*/*Sparc* and *Nsf* (nephrogenic system fibrosis), was observed in the mutant (Figure 1B).

Consistent with the increased apoptosis of NPCs associated with *Eya1*-deficiency (4), we observed increased expression of programmed cell death ligand-1 *PD-L1/Cd274* and reduced expression of anti-apoptotic factors including *Traf1* (TNF receptor-associated factor) (Supplementary Table S1) and *Cd5l/ATM* (apoptosis inhibitor of macrophage) (Figure 1B, Supplementary Table S1). Downregulation of *Spink4* (its downregulation known to be associated with poor survival of cancer cells), *Ttll9* (involved in microtubule cytoskeleton organization and protein polyglutamylation), and *Nppa* (has a role in mediating cardio-renal homeostasis) was also observed (Figure 1B). Among cell cycle regulators (Figure 1E), we detected upregulation of *Cdkn1c—*a potent tight-binding inhibitor of the G1 cyclin/CDK complexes, but downregulation of several cell cycle positive regulators, including *Ccno* and *Ccnd1/Ccne2/Cdc25a* necessary for G1/S phase transition, *Trim71* (E3 ubiquitin ligase that binds miRNA during G1/S transition), *E2f3/Plk1* (involved in G1/S phase transition), DNA repair proteins *Msh2/Rad51/Rad21l1/Rif1* and *Smchd1* (non-canonical member of the structural maintenance of chromosomes protein family). We also detected decreased expression of other regulators related to cell cycle progression/DNA replication/DNA repair-cell death, such as the ATP-dependent RNA helicase *Dhx9* (have a role in DNA replication and cell cycle progression) (38), *Mybbp1a* (the Myb proto-oncogene binding protein), and *Hmga2* (functions in cell cycle regulation through Ccna2) (39). Additionally, several chromatin remodeling factors, including components of the SWI/SNF complex *Smarcl1/Smarcc1/Smarca5*, the Polycomb group protein *Phf19*, the core histone macroH2A1 *H2afy* and *Bahcc1* (binds H3K27me3) (40), displayed decreased expression in *Eya1*cKO. This suggests that deletion of *Eya1* may lead to changes in overall chromatin accessibility. Nonetheless, the downregulation of these genes in *Eya1*cKO suggests that Eya1 may act as a transcriptional coactivator to positively regulate their expression in NPCs to maintain the cellular state.

We next clustered RNA-seq data for all DEGs into five groups using *K*means clustering analysis and performed Gene Ontology (GO) analysis (Supplementary Figure S1A). Downregulated DEGs enriched for ‘biological process’ related to ureteric development/kidney morphogenesis, nucleic acid metabolic process, chromosome organization and regulation of gene expression, while upregulated DEGs enriched for terms related to renal filtration cell differentiation/glomerulus development/cell differentiation involved in kidney development, nervous system development, cell migration/adhesion, ECM organization, and cardiac chamber formation. GO enrichment analysis of ‘molecular function’ revealed association of downregulated DEGs with nucleic acid/DNA/chromatin/histone/protein-containing complex binding and ATP hydrolysis/nucleoside triphosphatase/transcription regulator activity (Supplementary Figure 1B), while the upregulated genes were enriched for terms related to ECM/signaling receptor/protein-containing complex/integrin/PDGF/SMAD binding. These analyses provide insights into the possible biological processes regulated by Eya1 during kidney development and the potential molecular functions of its downstream genes. Collectively, our results show that Eya1 is necessary for the expression of numerous genes essential for the NPC maintenance and suggest that *Eya1* deletion may lead to abnormal activation of non-NPC or disease-related genes.

### Eya1 preferentially occupies promoter sequences and REST/NRSF recognition sites

**Figure 2.**
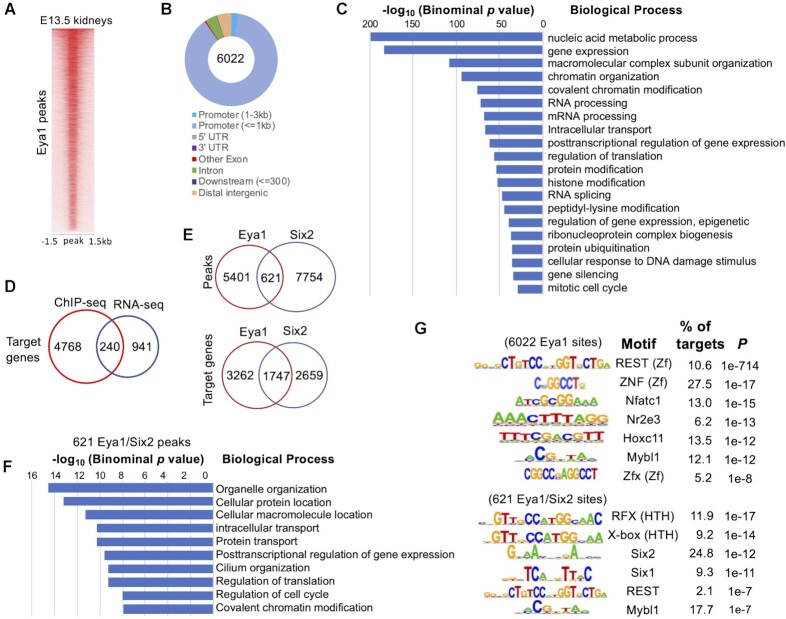
Genome-wide occupancy by Eya1 in E13.5 kidneys. (**A**) Heat map showing Eya1 peaks within a ±1.5-kb window centered on all Eya1 peaks in *Eya1^HA-FLAG^* kidneys. (**B**) Pie charts (Galaxy toolkits) showing genomic distribution. UTR, untranslated region. (**C**) GREAT analysis showing association of Eya1-enriched regions with terms in GO database. (**D**) Venn diagram indicating overlap of Eya1 putative target genes and DEGs regulated by Eya1. (**E**) Venn diagram indicating overlap of Eya1 peaks and Six2 peaks in E13.5 kidneys (from the public database GSE185050) (18) or Eya1 target genes and Six2 target genes. (**F**) GREAT analysis showing association of the 621 peak regions co-occupied by Eya1/Six2 with terms in GO database. (**G**) Sequence logos of the significantly enriched top motifs from Homer motif analysis, letter size indicates nucleotide frequency.

Eya1 has been considered a transcriptional coactivator, and it physically interacts with Six2 and Myc proteins in the kidney (4). To investigate the transcriptional network coregulated by Eya1-Six2, we performed ChIP-seq to profile the genomic occupancy feature of Eya1 in E13.5 kidneys to avoid epithelized nephron structures and compared it to our recent Six2 ChIP-seq dataset in E13.5 kidneys (18). For Eya1 ChIP-seq, we used an anti-FLAG antibody and chromatin isolated from E13.5 kidneys of *Eya1^HA-FLAG^* (2×HA-3×FLAG-Eya1) knockin mice that had previously been verified the expression of the multi-tagged Eya1 protein by using anti-FLAG or -HA antibodies (18). In peak calling against both genomic input and IgG controls, we identified 6022 Eya1 peaks from two datasets (Figure 2A, Supplementary Table S2). Analysis of their genomic distribution revealed that only ∼9% of Eya1 peaks were intronic and intergenic, while ∼90% of the peaks were located in proximal-promoter regions (Figure 2B). This suggests that Eya1 may be part of a regulatory complex composed of general TFs, RNA polymerase and other factors that control transcription initiation.

Consistent with the GO terms enriched in downregulated DEGs (Supplementary Figure S1) and its role in gene expression, DNA damage repair, cell cycle, signaling pathways, cancer metastasis (5,6), and ubiquitin-dependent proteasome pathway (41), GO analysis of Eya1 target genes for ‘biological processes’ and identified the top enriched terms related to nucleic acid metabolic process, gene expression, macromolecular complex subunit/chromatin organization, covalent chromatin/protein/histone modification, RNA processing/splicing, posttranscriptional regulation of gene expression, epigenetic regulation of gene expression (positive and negative), gene silencing, regulation of translation, ubiquitin-dependent/proteasome protein catabolic process, ribonucleoprotein complex biogenesis, cellular response to DNA repair, and mitotic cell cycle were overrepresented in Eya1 targets (Figure 2C). Analysis of GO molecular function showed significant enrichment for nucleic acid/RNA/chromatin binding (Supplementary Figure S2A), while terms related to TF activity/binding, transcription cofactor binding, ubiquitin-like protein ligase binding/ubiquitin-like protein transferase activity, and histone binding were also detected. Together, these analyses provide insights into how the initial Eya1 expression is diversified and amplified through its target genes to regulate diverse biological processes to maintain the nephron progenitor population.

Comparison with the RNA-seq data identified that 240 DEGs (136 downregulated and 104 upregulated) were bound by Eya1 (Figure 2D), suggesting that they are direct targets of Eya1. Pathway analysis of the 136 downregulated genes identified significantly enriched terms related to biological process of chromosome organization and negative regulation of gene expression or pathway related to microRNA in cancer (Supplementary Figure S2B), whereas no pathway was associated with the 104 upregulated genes. Nonetheless, these analyses further indicate that the Eya1 is involved in chromosome/chromatin organization.

Next, we compared the putative target genes/peaks of Eya1 with Six2 and identified 1747 genes (34.9% of Eya1 targets) and 621 peaks (10.3% of Eya1 peaks) shared by Eya1 and Six2 (Figure 2E). Among these peaks, only ∼3% were located in intronic and intergenic regions, and the majority were in proximal-promoter areas (Supplementary Figure 2C). GO annotation indicated that the 621 Eya1/Six2 peaks were associated with genes related ‘biological process’ of organelle organization, cellular protein/macromolecule location, intracellular/protein transport, posttranscriptional regulation of gene expression, cilium organization, regulation of translation/cell cycle and covalent chromatin modification (Figure 2F), but no nephron-related term was enriched. GO analysis of ‘cellular component’ displayed enrichment for terms related to nucleoplasm, microtubule organizing center, centrosome, ribonucleoprotein complex, cilium, ciliary transition zone, nucleolus and chromatin (Supplementary Figure S2D).

We then searched for the binding motif of Six2 in the Eya1 peaks. To our surprise, the most significantly enriched motif was the binding motif of the zinc-finger transcriptional repressor REST (RE1 silencing TF, also called NRSF), which is present in 10.6% of Eya1-occupied sites (Figure 2G). This is consistent with the GO terms ‘gene silencing’ and ‘negative epigenetic regulation of gene expression’ associated with Eya1-occupied sites (Figure 2C). DNA recognition sequences for other zinc-finger proteins such as ZNF (27.5% of Eya1 peaks) and Zfx (5.2% of Eya1 peaks), nuclear factor Nfatc1, and nuclear receptor Nr2e3, Hoxc11 and proto-oncogene Mybl1 were also found (Figure 2G). Of the 621 Eya1/Six2 sites, 24.8% (154 sites) contained binding motifs for Six2. Therefore, only ∼2.6% of Eya1-occupied sites have Six2 recognition sequences. Interestingly, 2.1% of the 621 Eya1/Six2 sites also had REST recognition sequences, suggesting that Six2 may cooperate with Eya1 to function in a REST-dependent gene repression network to maintain the identity of NPCs. Thus, this genome-wide analysis collectively provides insight into potential DNA-binding proteins with which Eya1 interacts to target CREs and regulate gene expression. Since the motifs of zinc-finger proteins have the highest proportion (∼43.3%) in the Eya1 peaks, Eya1 may preferentially interact with different zinc-finger proteins to bind the motif sequences. Identification of the REST-binding motif as the most significantly enriched motif at Eya1-occupied sites indicates that Eya1 physically associates with REST-dependent transcriptional complexes to actively repress or silence gene expression.

### Approximately 86% of Eya1-occupied sites are associated with H3K27ac-deposition, and 76% of Eya1-occupied sites without H3K27ac-deposition possess the REST motif

**Figure 3.**
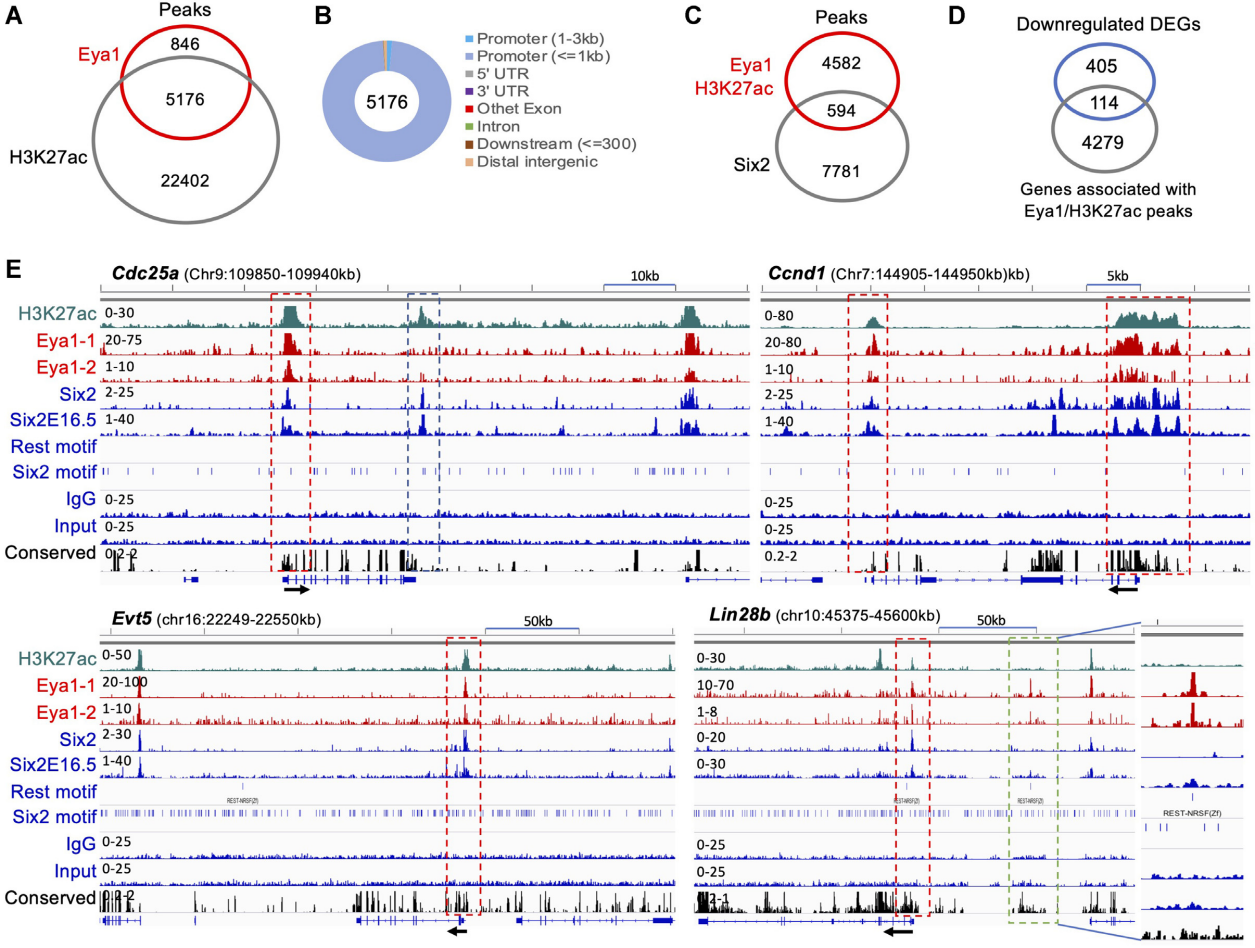
Eya1 occupies preferentially promoter sequences associated with H3K27ac-deposition. (**A**) Venn diagram indicating overlap of Eya1 peaks with H3K27ac in E13.5 kidneys (GSE185050). (**B**) Genomic distribution of Eya1 peaks associated with H3K27ac-deposition. UTR, untranslated region. (**C**) Venn diagram indicating the overlap of Eya1/H3K27ac peaks and Six2-binding sites. (**D**) Venn diagram indicating the overlap of Eya1/H3K27ac peaks and downregulated DEGs in *Eya1*cKO. (**E**) Genomic browser visualization of overlapping occupancy of H3K27ac, Eya1, Six2 in E13.5 kidneys and E16.5 kidneys. Six2E16.5 is from the public database (GSE39837) (68). Dashed boxes indicate DNA sequences occupied by Eya1/Six2 (red), Six2 with H3K27ac-deposition (blue) and Eya1 alone (green). The direction of transcription is indicated by the arrow beginning at the transcription start site.

As most Eya1 peaks are located in the promoter region, we anticipated these peaks to be associated with the transcriptionally active histone mark H3K27ac. Indeed, compared with H3K27ac ChIP-seq data from E13.5 kidneys (18), 5176 (∼86%) Eya1-occupied sites were found to be associated with H3K27ac (Figure 3A), confirming that Eya1 is involved in transcriptional activation. Of the 5176 peaks, ∼99% were located in proximal-promoter regions, and only ∼1% were located in intronic and intergenic regions (Figure 3B), while 594 sites (11.5%) were also occupied by Six2 (Figure 3C). In comparison with the 136 downregulated Eya1 targeted DEGs in *Eya1*cKO, we found that 114 genes (83.8%) associated with H3K27ac-deposition, of which 54 (47.4%) were also targeted by Six2 (Figure 3D), indicating that these genes are positively regulated by Eya1 or Eya1/Six2. These genes include *Six2*, *Pax2*, *Gdnf, Bahcc1, Cdc25a, Ccnd1, Ccne2, E2f3, Lin28b, Fgfr1, Chd7, H2afy, Hdac2/Dmnt1, Hmga2, Hoxa11/Hoxd11, Mybbp1a, Rif1, Sall1, Smc4, Smarcc1* and *Sox11*. For example, at the locus of the cell cycle regulator *Cdc25a* (Figure 3E), genomic browser visualization showed that Eya1/Six2 co-occupied the promoter, while Six2 alone also occupied the 3’ downstream CRE/enhancer. Co-occupancy of Eya1/Six2 to regions flanking the *Ccnd1* gene and promoters of *Evt5* (Figure 3E), *Ccne2, E2f3*, *Hmga2* or *Dnmt1* (Supplementary Figure S3) was observed. Interestingly, at the locus of *Lin28b* or *Pax2*, Eya1/Six2 co-occupied their promoter sequences, but Eya1 alone also associated with a conserved intergenic CRE ∼60-kb upstream of the *Lin28b* promoter or ∼53-kb upstream of the *Pax2* promoter, both containing a REST motif without H3K27ac-deposition (Figure 3E, Supplementary Figure S3). Thus, while Eya1 and Six2 are involved in the transcriptional activation of a set of genes encoding proteins required for NPC maintenance, Eya1 may simultaneously cooperate with other partners to assemble regulatory complexes on the REST recognition sequences to actively repress the expression of specific genes such as *Lin28b* and *Pax2* to balance their expression levels.

**Figure 4.**
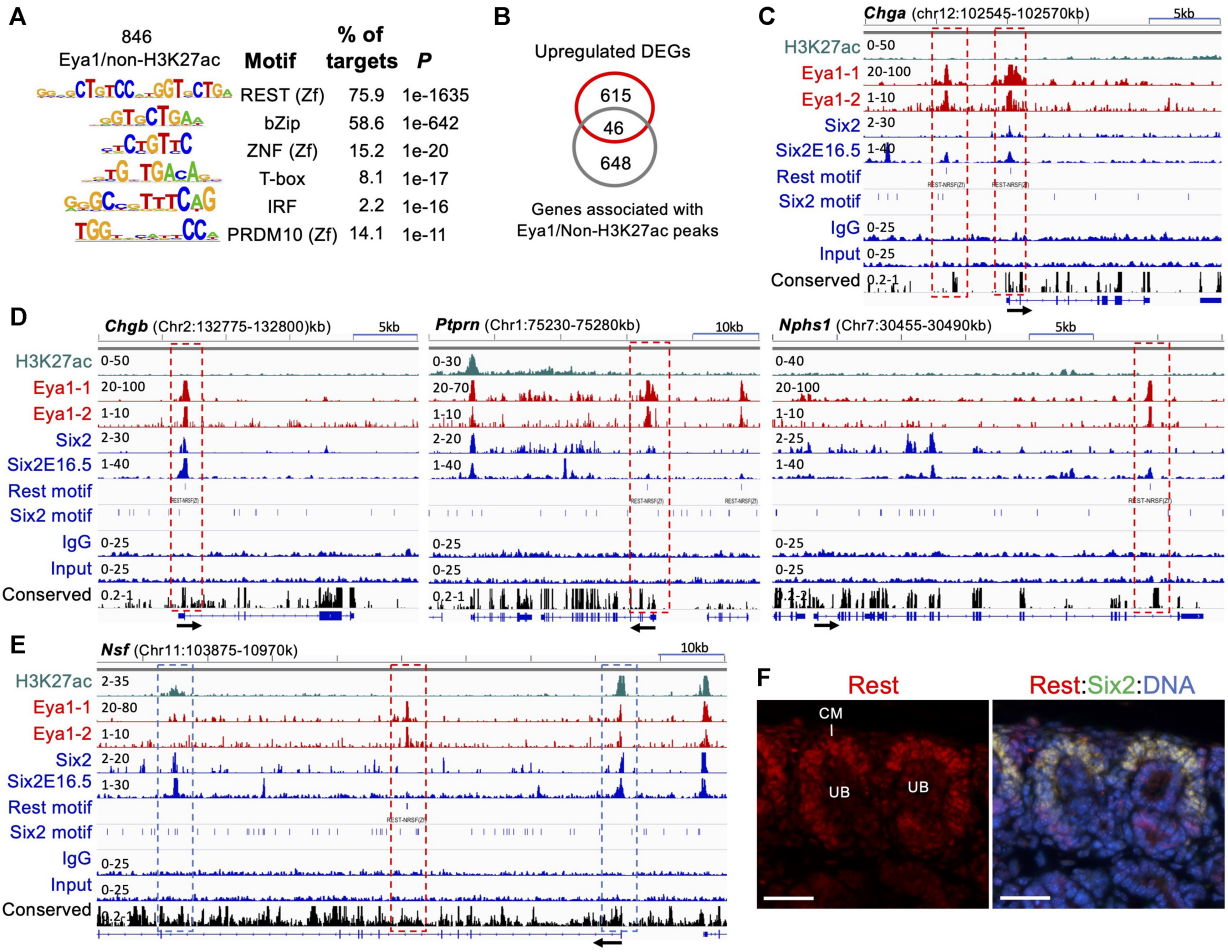
Approximately 76% of Eya1-occupied sites without H3K27ac-deposition possess REST-binding motif. (**A**) Sequence logos of the significantly enriched top motifs of the 846 non-H3K27ac/Eya1-occupied sites, letter size indicates nucleotide frequency. (**B**) Venn diagram indicating the overlap of Eya1/non-H3K27ac peaks and Eya1 target genes updated in *Eya1*cKO. (**C–E**) Genomic browser visualization of occupancy of H3K27ac, Eya1, Six2 in E13.5 kidneys and Six2 in E16.5 kidneys. The direction of transcription is indicated by the arrow beginning at the transcription start site. Boxes outlined by red dashed line indicate Eya1 peaks with REST-binding motif (C-E), while blue boxes indicate Six2 peaks without REST-binding motif. (**F**) Co-immunostaining for anti-Rest (red) and Six2 (green) showing higher expression levels of Rest in Six2^+^ cap mesenchyme (CM) surrounding ureteric bud (UB). Scale bar: 30 μm.

To further explore the potential role of Eya1 and its association with REST recognition sequences in transcriptional repression during kidney development, we searched the 846 Eya1/non-H2K27ac peaks for TF binding motifs. Notably, the most significantly enriched motif was the REST-binding motif, present in ∼76% of these peaks (Figure 4A). DNA binding motifs for bZip proteins, zinc-finger proteins ZNF and PRDM10 (PR domain zinc-finger), T-box and IRF proteins were also identified (Figure 4A). Of the 694 genes associated with the 846 Eya1/non-H3K27ac peaks, 46 genes (44.2%) were among the 104 upregulated Eya1 targeted DEGs (Figure 4B). They include numerous genes associated with kidney diseases, such as *Chga/Chgb*, *Ptprn*, *Nphs1/Nphs2*, *Nsf*, *Rph3a*, *Podxl* and *Cyp11a1*. As revealed by genomic browser visualization (Figure 4C), Eya1 showed strong enrichment at the REST recognition sequences of the *Chga* promoter and a conserved region ∼4-kb upstream of the promoter; neither was associated with H3K27ac-depositon and there was only some residual Six2 signal. At the loci of *Chgb* (Figure 4D) and the podocyte-specific gene *Rph3a* (Supplementary Figure S4A), Eya1/Six2 occupied REST recognition sequences at their promoter regions without H3k27ac-depositon. We also observed Eya1-occupancy to the proximal intronic REST elements of *Ptprn* and the podocyte-specific gene *Nphs1*, respectively, both of which are highly conserved and lack Six2-binding and H3K27ac-deposition. (Figure 4D). While the promoter of the *Nsf* (nephrogenic system fibrosis) gene was co-occupied by Eya1/Six2, Six2 alone also bound to a distal intronic CRE/enhancer ∼+70-kb, and Eya1 alone occupied another distal intronic CRE ∼+35-kb without H3k27ac-deposition but with two REST motifs (Figure 4E). At the podocyte-specific gene *Podxl*, a highly conserved REST element at ∼150-kb upstream of the promoter without H3K27ac-deposition was co-occupied by Eya1/Six2 (Supplementary Figure S4A). Eya1-occupancy to a distal intronic REST element ∼+15-kb of the proximal tubule gene *Slc13a3* was also observed (Figure S4A). Thus, Eya1-occupancy to REST-binding sequences present in CERs of target genes without H3K27ac-deposition and their increased expression in *Eya1cKO* strongly suggest that Eya1 cooperates with a REST-dependent gene repression network to negatively regulate the transcription of these genes to prevent NPCs from differentiating into other cell types.

Gene repression by the master regulator REST relies on the recruitment of multiple enzymatic corepressor complexes that modify chromatin to repress transcription (42). It has been shown to play roles in regulating pluripotency and self-renewal of embryonic stem cells (43). Mutations in the *REST* predispose to Wilms tumor, which accounts for ∼2% of Wilms tumor (44). A recent study showed that REST protects the kidney from injury and degeneration during aging (45). Since it was unclear whether the Rest protein was expressed in NPCs, we next examined the presence of Rest expression in kidneys. Genomic visualization revealed Eya1/Six2 co-occupancy to the *Rest* promoter associated with H3K27ac-depostion (Supplementary Figure S4B), suggesting that *Rest* is transcriptionally active in kidneys and is co-regulated by Eya1/Six2. We used an anti-Rest antibody previously used by others (46,47) to immunostain mouse kidney sections and found that the expression level of Rest was higher in Six2^+^ NPCs than in other kidney structures (Figure 4F, Supplementary Figure S4C). Altogether, these results provide the first basis for linking Eya1 to a REST-dependent gene repression network to repress the expression of genes encoding non-NPC signature proteins. Our findings demonstrate that Eya1 acts as both a transcriptional activator and a repressor, simultaneously activating or repressing the expression of different genes to maintain the cellular state of nephron progenitors.

### Mass spectrometry analysis identifies Eya1-interaction network involved in transcriptional, posttranscriptional and translational regulation

**Figure 5.**
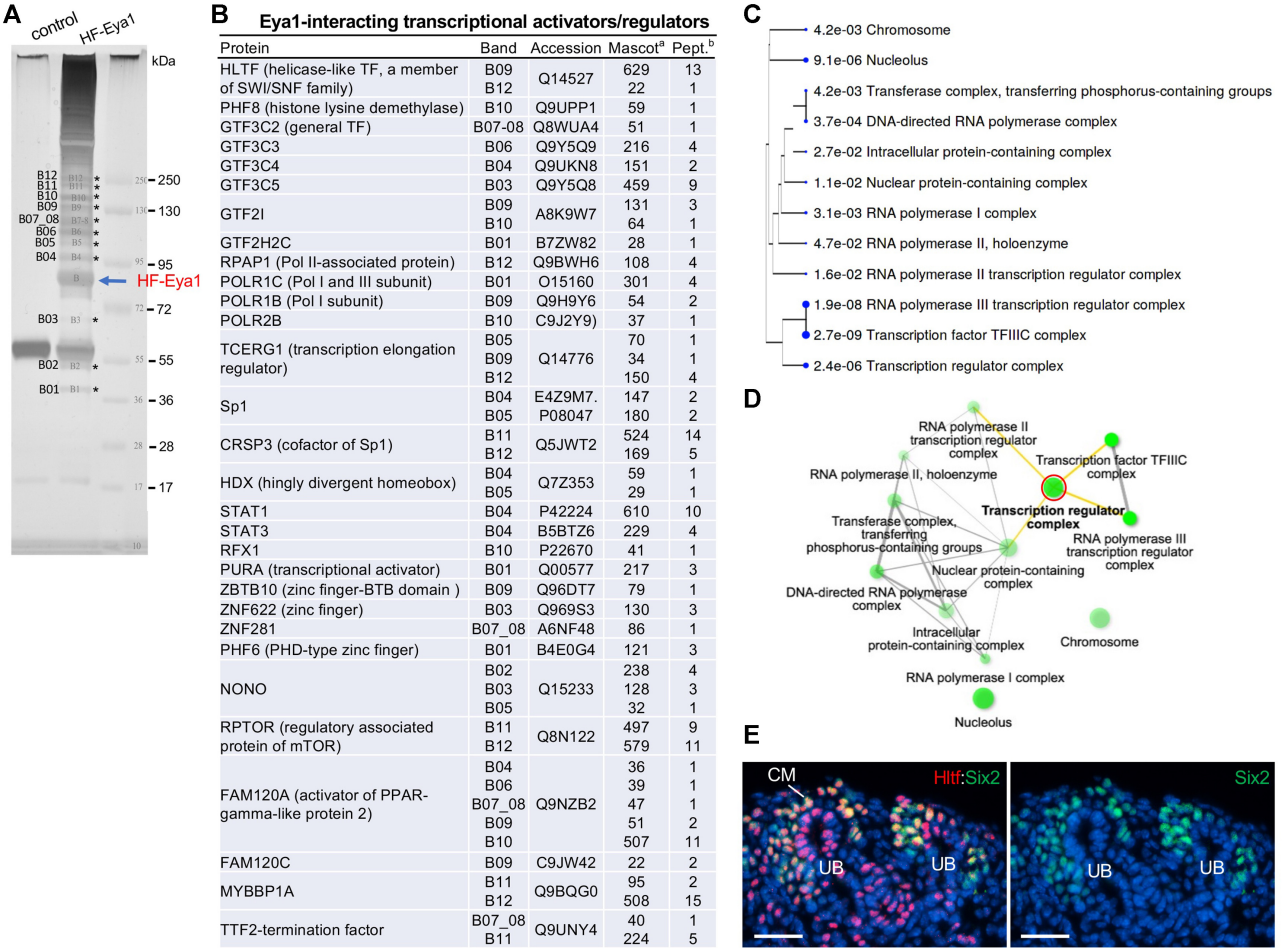
Purification of Eya1 and its interacting proteins. (**A**) Silver stained NuPAGE of a HA-FLAG-Eya1 (HF-Eya1) and control purification. The HF-Eya1 band is indicated. The asterisks (B1–B12) indicate bands used for mass spectrometry analysis. (**B**) List of Eya1-interacting transcription factors/activators as identified by mass spectrometry analysis of purified Eya1 samples. ^a^Mascot score for the specified protein in the Eya1 sample. ^b^Number of identified unique, nonredundant peptides for the specified protein in the Eya1 sample. (**C**) A hierarchical clustering tree summarizing the correlation among significant pathways identified by GO enrichment analysis for cellular component. Pathways with many shared genes are clustered together. Bigger dots indicate more significant *P*-values. (**D**) An interactive plot showing the relationship between enriched pathways for cellular component. Darker nodes are more significantly enriched gene sets and bigger nodes represent larger gene sets. Thicker edges represent more overlapped genes. The red circle indicates the ‘transcriptional regulation complex’ centered pathway and its relationship with other related pathways (yellow lines). (**E**) Coimmunostaining for anti-Hltf (red) and -Six2 (green) on sections of E17.5 kidneys. Abb.: CM, cap mesenchyme; UB, ureteric bud. Scale bar: 30 μm.

Eya1 does not directly bind to DNA and its transcriptionally active or repressive function depends on its interacting partners. We searched for known Eya1 interaction networks using inBio Discover^TM^ or String tool and found 26 or 10 Eya1-interacting proteins (Supplementary Figure S5A, B). These known proteins include TFs SIX1/SIX2/SIX3/SIX4/SIX5, DACH1, SOX2, NEUROD1, NEUROG1, MYC, TCF7L2, components of the SWI/SNF chromatin-remodeling factors, EYA3/RBCK1, apoptosis and DNA repair factors ATM/H2AX, proteasome-related factors FZR1 and FBXW7, and G-proteins. Since none of these known Eya1-interacting proteins has been shown to be involved in REST-regulatory complexes or basal transcription components, to gain more insight into the transcriptional network in which Eya1 operates, we sought to use mass spectrometry to identify Eya1-interacting proteins. In a HEK293 stable cell line expressing 3 × FLAG-Eya1, we have shown that Eya1 is regulated throughout the cell cycle and is more abundant in mitotic cells, similar to the endogenous Eya1 expressed in the C2C12 mouse myoblast cell line and mouse embryonic fibroblast cells (41). We thus generated another stable HEK293 cell line that expresses the 2× HA-3× FLAG-Eya1 (HF-Eya1) protein, purified HF-Eya1 from the cell extracts by a tandem affinity purification protocol to reduce background (first with anti-FLAG and then with anti-HA to purify FLAG-elute, see Methods) and analyzed 12 bands that were well separated and identifiable on the SDS-PAGE gel (B1–B12) using mass spectrometry (Figure 5A). In addition to multiple components of the SWI/SNF chromatin remodeling complexes co-purified by Eya1 (18), we identified 30 Eya1-interacting factors that were involved transcriptional activation (Figure 5B). Of them, we identified TFs with enzymatic activity such as the helicase-like TF HLTF (a new member of the SWI/SNF family) and the histone lysine demethylase PHF8 (PHD-type zinc-finger protein) and PHF6. Eya1 co-purified several general TFs (components of GTF2 and GTF3), subunits of RNA polymerase I, II and III and the transcription elongation regulator TCERG1 (Figure 5B). This is in line with our genome-wide analysis, which identified 90% of Eya1-occupied sites at promoter regions.

Consistent with the identified binding motifs of zinc fingers and RFX/X-box at the Eya1 peaks (Figure 2G), several classes of zinc-finger proteins, including PHF8/PHF6, Sp1 and its cofactor CRSP3, ZBTB10, ZNF281 and ZNF622, and the RFX/X-box TF RFX1 were present in Eya1 immunoprecipitants (Figure 5B). Additionally, Eya1 copurified the signal transducer and activator STAT1/STAT3, the highly divergent homeobox protein HDX, the transcriptional activator PURA, the non-POU domain-containing octamer-binding protein NONO, RPTOR (mTOR associated regulatory protein), FAM120A (constitutive activator of PPAR-gamma-like protein), and the transcription termination factor TTF2. As outlined in the hierarchical clustering tree (Figure 5C) or interactive plot (Figure 5D), GO enrichment analysis of the cellular component network of the factors listed in Figure 5B revealed the most significantly enriched pathways and their relationship or correlation between RNA polymerase I, II and III complexes, transcription regulator complex, DNA-directed RNA polymerase complex, transferase complex, and nucleus. Factors involved in RNA processing, posttranscriptional and translational regulation were also detected (Supplementary Table S3, Supplementary Figure 5C). Therefore, the association of these proteins with Eya1 suggests that they may serve as interaction partners of Eya1 in gene transcriptional, posttranscriptional and translational regulation.

We further investigated whether the helicase protein Hltf is expressed in the NPCs during kidney development. Immunostaining detected a broad expression pattern of Hltf in multiple cell types in E17.5 kidneys, including Six2^+^ NPCs, branching UBs and stromal cells (Figure 5E). Together, our results suggest that Eya1 may cooperate with a variety of chromatin remodeling factors and different specialized DNA-binding factors to regulate chromatin structures and promote the assembly of various large-scale regulatory complexes on Eya1-occupied promoter sequences and CREs/enhancers.

### Copurification of cell cycle regulators and DNA replication and repair proteins by Eya1

**Figure 6.**
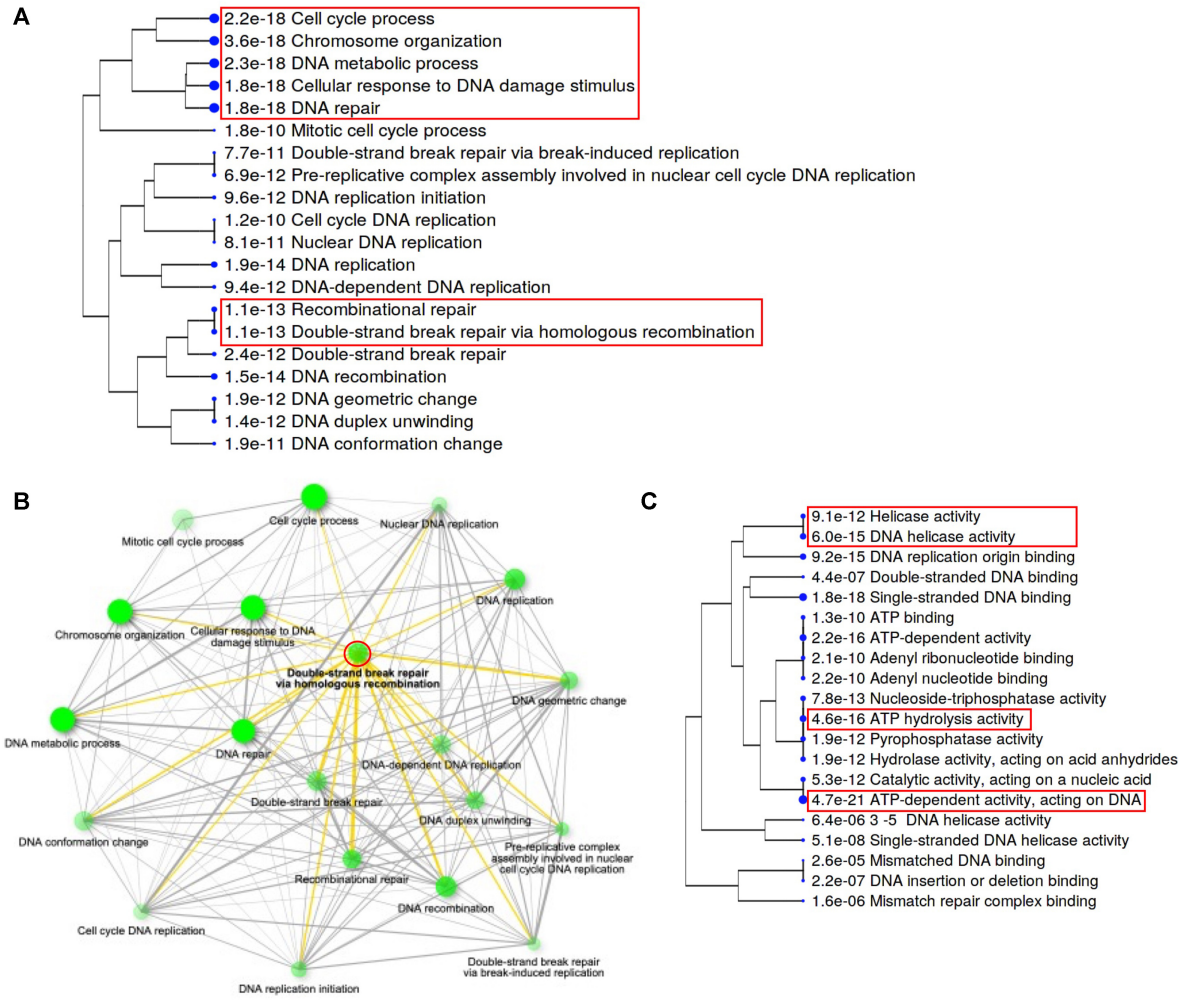
GO analysis of all proteins listed in Table 1. (A, C) A hierarchical clustering tree summarizing the correlation among significant pathways for biological process (**A**) or cellular component (**C**) identified by GO enrichment analysis. Pathways with many shared genes are clustered together. Bigger dots indicate more significant *P*-values. The red box in A indicates pathways that are significantly enriched or associated with recombinational repair, while in C indicates ‘cellular component’ associated with helicase or ATPase activity. (**B**) An interactive plot showing the relationship between enriched pathways for biological process. Darker nodes are more significantly enriched gene sets and bigger nodes represent larger gene sets. The red circle indicates the ‘double-strand break repair via homologous recombination’ centered biological process and its relationship with other related pathways (yellow lines). Thicker edges represent more overlapped genes.

Previous studies showed that EYA dephosphorylates γ-H2AX (23,48), which is required for H2AX to recruit DNA repair proteins to the damaged site during DNA double-strand break response (23,49). Consistent with this, in Eya1 immunoprecipitants (Table 1), in addition to cell cycle/apoptosis-related regulators, we detected DNA replication and repair proteins such as the DNA replication factor RFC1, the MCM replication licensing family, various cohesion factors including the cohesion complex SMC family—key regulators of DNA repair, chromosome condensation and chromosome segregation from bacteria to humans (50), DNA mismatch repair proteins MSH2/MSH3/MSH6, double-strand break repair proteins RAD21/RAD50, and members of several other repair/cell death-interacting factors. Since these factors are critical for efficient homologous recombination-mediated DNA double-strand break repair that requires the sister chromatid as a template for high-fidelity repair (51,52), Eya1 may cooperate with these factors to regulate DNA double-strand break repair pathway by homologous recombination. Indeed, among the important pathways associated with all proteins listed in Table 1 (Figure 6A, B), we noticed significant enrichment of biological processes related to recombinational repair/double-strand break repair via homologous recombination. Notably, however, enrichment analysis of molecular functions identified significant pathways related to ATP-dependent activity acting on DNA and ATP hydrolysis/helicase/DNA helicase activity (Figure 6C).

**Table 1.** Eya1-interacting factors involved in cell division and DNA repair

Protein	Band	Accession	Mascot^a^	Pept.^b^
**DNA replication and repair proteins**				
RFC1 (replication factor, DNA-dependetn ATPase)	B11 B12	P35251	32 367	1 6
MCM3 (DNA replication licensing factor)	B05 B06	P25205	191 248	5 6
MCM4	B05 B06	B3KMX0	44 161	1 3
MCM5	B04	B1AHB1	399	7
MCM6	B05 B06	Q14566	29 191	1 5
MCM7	B04	P33993	90	2
MCM8	B05	E7EQU7	56	2
MCM10	B07_08	Q5T670	35	1
SMC1A (structural maintenance of chromosomes)	B11 B12	Q14683	24 788	1 18
SMC2	B11 B12	O95347	2185 41	35 1
SMC3	B11 B12	Q9UQE7	2024 441	33 10
SMC6	B10	C9JMN1	53	1
PDS5A (sister chromatid cohesion protein)	B11 B12	Q29RF7	45 626	2 14
CHTF18 (chromosome cohesion factor)	B07_08 B09	Q8WVB6-2	50 828	1 22
WAPAL (regulator of sister chromatid cohesion)	B12	B2RTX8	314	6
NCAPG (condensin complex subunit)	B07_08 B09	Q9BPX3	29 747	1 14
Condensin complex subunit 2	B05	A8K4T8	420	7
MSH2 (DNA mismatch repair protein)	B07_08	P43246	78	3
MSH3	B10	P20585	31	1
MSH6	B07_08 B12	B4DF41	108 756	2 13
RAD21 (double-strand-break repair protein)	B6	O60216	202	5
RAD50	B12	Q92878	73	2
MMS19-like (nucleotide excision repair protein)	B06 B07_08	B4DQX2	92 39	2 1
PDCD6IP (programmed cell death 6-interacting protein)	B04 B05 B06 B07_08	Q8WUM4	279 150 1176 46	6 2 17 1
KIAA0776 (E3 UFM1-protein ligase 1, DNA damage respinse)	B04	O94874	218	6
BCCIP (BRCA2 and CDKN1A inetracting protein)	B01	B3KP45	63	1
**Cell cycle and apoptosis regulator**				
CCAR2 (KIAA1967, cell cycle and apoptosis)	B09	Q8N163	1032	20
CIP2A (inhibits PP2A and stabilizes Myc in cancer)	B05	Q8TCG1	456	13
CDC27 (cell division cycle protein)	B05	P30260	107	2
PCID2 (cell survival and cell-cycle)	B01	A6NC39	94	2
TAB1 (proliferation and apoptosis)	B03	Q15750	107	3
MAGED1 (inhibits cell cycle and facilitates NGFR-mediated apoptosis)	B03 B04 B05	Q9Y5V3-2	73 118 1199	2 2 15

^a^Mascot score.

^b^Number of peptide.

Additionally, Eya1 copurified E3 ubiquitin ligase, 26S proteasome regulatory subunits and the MAP kinases (Supplementary Figure S6A), which were strongly enriched for pathways related hematopoietic stem cell differentiation, activation of the innate immune response, cell cycle G2/M phase transition and the MAPK cascade (Supplementary Figure S6B). This finding is also consistent with the previous report that EYA’s threonine-phosphatase activity modulates innate immune responses (53) and that Eya family proteins are located both in the cytoplasm and nucleus (6,54).

The Eya protein family has no nucleus localization signal and it requires cofactors to mediate its nuclear location. In the NPCs, Eya1 is predominantly located in the nucleus, and its nuclear location is mediated through interaction with Six2 as Eya1 is localized in the cytoplasm of *Six2^−^^/^^−^* NPCs (4,14). Interestingly, our mass spectrometry analysis identified several importins and exportins (Supplementary Table S4), suggesting that these importins and exportins may be involved in transporting Eya1 between the cytoplasm and nucleus. Together, these analyses provide insight into the underlying molecular mechanisms that mediate the subcellular localization of Eya1 and how it regulates cell cycle progression, DNA replication and repair.

### Identification of HDAC1 and REST corepressors as Eya1-interacting proteins

**Figure 7.**
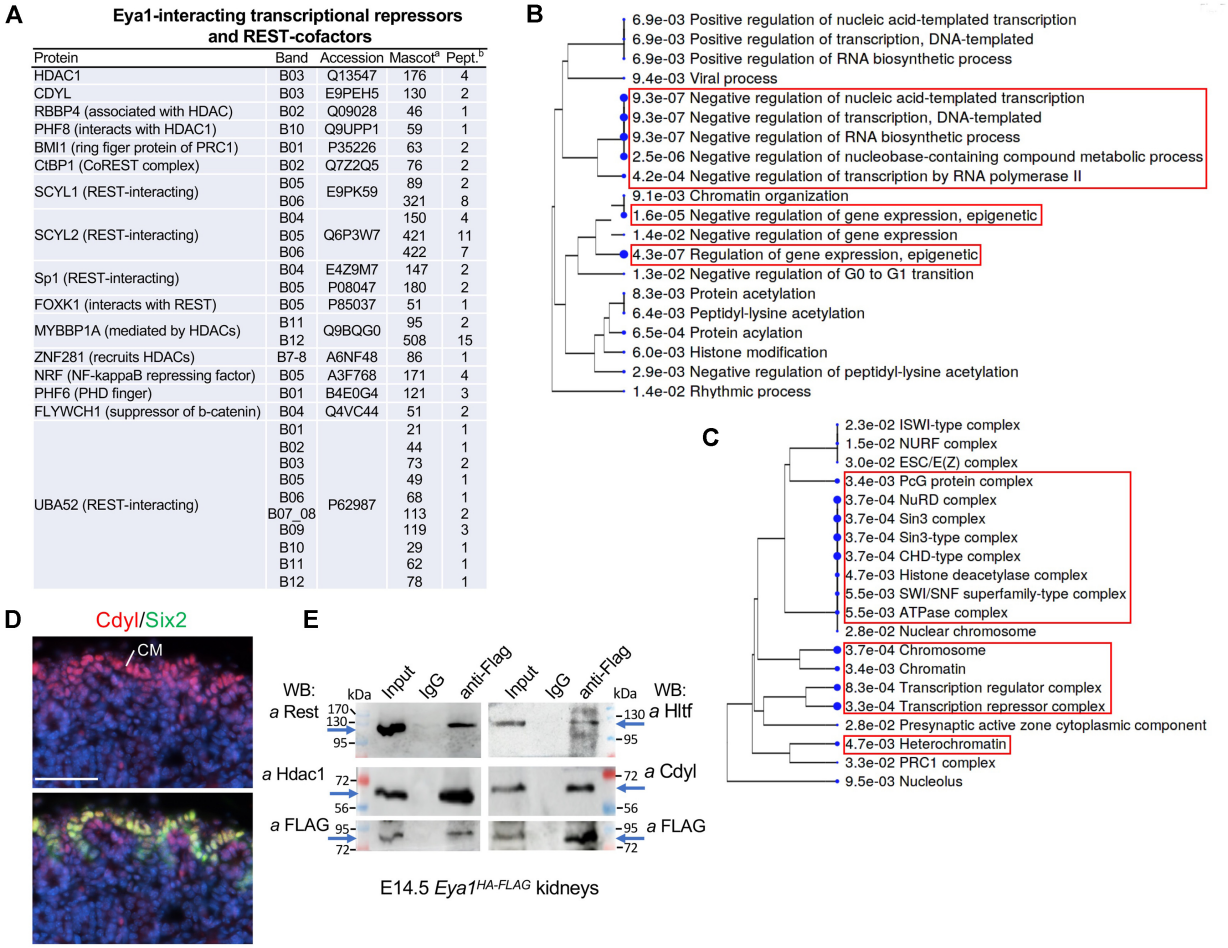
Identification of REST-interacting proteins in Eya1 immunoprecipitates. (**A**) List of Eya1-interacting REST-interacting chromatin remodeling factors and corepressors/proteins identified by mass spectrometry analysis of purified Eya1 samples. ^a^Mascot score for the specified protein in the Eya1 sample. ^b^Number of identified unique, nonredundant peptides for the specified protein in the Eya1 sample. (**B**, **C**) Hierarchical clustering tree summarizing the correlation among significant pathways for biological process (B) or molecular function (C) identified by GO enrichment analysis. Pathways with many shared genes are clustered together. Bigger dots indicate more significant *P*-values. The red boxes indicate top enriched ‘biological processes’ in B or ‘molecular functions’ in C. (**D**) Coimmunostaining for Cdyl and Six2 showing higher levels of Cdyl expression in Six2^high^ cap mesenchyme (CM) and branching ureteric bud (UB). (**E**) Coimmunoprecipitation of anti-FLAG of cell extracts prepared from E14.5 *Eya1^HA-FLAG^* kidneys. Antibodies used for western blot detection were indicated. Eya1 co-immunoprecipitated Rest (∼122-kDa), Hdac1 (∼60-kDa), Hltf (∼114-kDa) and Cdyl (∼66-kDa). Scale bar: 50 μm.

Search for known REST-interacting proteins using inBio Discover™ tool revealed REST interaction networks containing 56 proteins (Supplementary Figure S7A). Multiple of these REST-interacting proteins were copurified by Eya1 (Figure 7A). Of them, we detected HDAC1 (histone deacetylase 1), CDYL – a chromodomain on Y-like protein and REST corepressor that physically bridges REST and the histone methylase G9a to repress transcription (55), RBBP4 (a chromatin remodeling factor implicated in transcriptional repression associated with histone deacetylation), PHF8 (histone lysine demethylase interacting with HDAC1), BMI1 (a component of Polycomb repression complex PRC1 associated with histone deacetylation), CtBP1 (a component of the REST corepressor complex), and the REST-interacting proteins SCYL1/2, Sp1, FOXK1 (forkhead box protein) and UBA52. We also detected MYBBP1A (proto-oncogene MYB-binding protein) (56) and ZNF281, which are both activators and repressors and their repressive function is mediated by HDACs. While ZNF281 is a component of a protein interaction network for pluripotency of embryonic stem cells (57), it also recruits HDACs to repress Nanog and has a role in DNA repair (58–60). Other repressors such as NRF, PHF6, and FLYWCH1 (a zinc-finger protein), which acts as a repressor of β-catenin (61), were also copurified by Eya1 (Figure 7A). Since REST mediates active repression via recruitment of HDACs by its corepressors (62), the physical association between Eya1, HDAC1 and REST-interacting proteins or corepressors further suggests that Eya1 may recruit HDAC1 to facilitate the assembly of protein complexes at the REST recognition sequences to actively repress or silence non-NPC lineage gene transcription.

GO ‘biological process’ analysis of all genes listed in Figure 7A indicated that the most significantly enriched pathways were related to negative regulation of transcription and epigenetic regulation/negative epigenetic regulation of gene expression (Figure 7B). The top enriched pathways for molecular function of these proteins were related to chromosome, transcription repressor/regulator complex, NuRD/Sin3/CHD (chromodomain helicase DNA-binding)-type complex (Figure 7C). Overall, these data are in line with the GO terms ‘gene silencing’ and ‘negative epigenetic regulation of transcription’ enriched at Eya1 peaks (Figure 2C) and the REST recognition sequences present in 76% of non-H3K27ac/Eya1-occupied sites (Figure 4A). These findings also point to roles for Eya1 in the epigenetic regulation of gene expression either positively or negatively through physical association with a variety of chromatin/nucleosome remodeling factors.

Since we only performed mass spectrometry on 12 bands excised from the gel (Figure 5A), it is unlikely that our analysis covered all Eya1-associated proteins in the Eya1 immunoprecipitates, which explains why REST was not detected in the mass spectrometry. Nonetheless, it is essential to use independent methods to verify the candidate proteins. Therefore, we next performed immunostaining and co-immunoprecipiation (coIP) to confirm the presence of the identified interactors Hdac1 and Cdyl in NPCs and physical interactions between Eya1 and Hdac1, Cdyl or Rest in the developing kidneys. Previous studies showed that Hdac1 and Hdac2 are highly expressed in the NPCs, branching UB and the stroma in the developing kidneys and deletion of both *Hdac1/Hdac2* results in small kidney size, lack of nephrogenic zone, nascent nephron and glomerulus, and formation of multiple cysts (63). Immunostaining for Cdyl revealed higher levels of its expression in Six2^+^ NPCs on the peripheral side of the branching UB, but its expression was downregulated in the differentiating progenitors migrating to the ventral side of the branching UB (Figure 7D, Supplementary Figure 7B). Its expression was also detectable in the branching UB tips. CoIP using extracts prepared from E14.5 kidneys of the multi-tagged *Eya1* knockin (*Eya1^HA-FLAG^*) embryos (18) demonstrated physical association between Eya1 and the Rest repressors Rest/Hdac1/Cdyl (Figure 7E). It also confirmed the physical association between Eya1 and the transcriptional activator Hltf (Figure 7E). Therefore, in conclusion, our tandem FLAG- and HA-affinity purification protocol identified novel, independently verifiable and biologically relevant proteins, expanding the Eya1 interaction networks. Since Eya1 is abundantly distributed in undifferentiated cells during development, and the EYA family is overexpressed in many cancer cell lines, our mass spectrometry data provide insights into various protein complexes that Eya1 may form with different factors to regulate cell proliferation and survival.

## DISCUSSION

EYA was initially identified as a transcriptional activator based on its transcriptional activation function in its NT domain (7). Since no intrinsic DNA-binding capacity of EYA has been found, and their conserved C-terminal ED interacts with DNA-binding proteins such as Six/So, it is generally believed that EYA is a transcriptional coactivator that relies on DNA-binding partner proteins to regulate gene expression. Subsequently, studies have shown that EYA has intrinsic threonine/serine and tyrosine phosphatase activity (64,65). In kidney development, Eya1 physically interacts with Myc and also dephosphorylates Myc at pT58 in the MM progenitors (4). Mice lacking the *Eya1* exhibit renal agenesis due to lack of MM formation (1,3), while *Eya1* deletion after UB outgrowth results in downregulation of *Six2* expression and depletion and premature differentiation of the NPCs (4). While these findings indicate a link between the acquisition of a nephron fate and Eya1 function, there is limited information on Eya1’s physiological substrates, downstream targets and Eya1-centered networks. In this study, we characterized Eya1 regulated genes, associated CREs and proteins in a combined and unbiased approach. Our findings indicate that Eya1 has a broad range of activities implicated in nucleosome organization and remodeling, activation and repression of gene expression, chromosome structure, cell cycle, and DNA replication and repair. Due to the lack of intrinsic DNA-binding activity, Eya1 probably works by forming unique multiprotein complexes, which help Eya1 exert its activity in a more efficient and specific manner in various fundamental biological processes.

Gene expression takes place within the confines of chromatin, where DNA is assembled into nucleosomes and associated with non-histone chromosomal proteins. Critical components of the transcriptional machinery (TFs, factors required for enhancer or silencer activity, and RNA polymerases) cannot interact with their target sequences unless they are accessible. Chromatin remodeling complexes regulate chromatin structure in an ATP-dependent manner to make chromatin available to proteins to access DNA. Our results show that Eya1 is broadly associated with various types of factors with ATPase or helicase activity (Figures 5, 6C and 7, Supplemental Table S3) and zinc-finger proteins that are known to be involved in chromatin remodeling. Thus, we speculate that Eya1-occupancy to CREs is connected to chromatin remodeling. Eya1 may belong to a class of ‘pioneer’ factors that can interact with nucleosomal DNA and induce a change in the configuration of nucleosomes to create nucleosome-free regions to promote the assembly of a variety of different large-scale regulatory complexes. Eya1-controlled remodeling of nucleosomes at promoters or CREs may depend on interaction with context-dependent and specialized DNA-binding partners that help create and maintain nucleosome-free regions of chromatin that are accessible to other functionally specific factors. One of such specialized DNA-binding partners is Six2 (4). Through interaction with the SWI/SNF remodelers and Six2, Eya1 recruits the SWI/SNF complex to Six2-occupied CREs that are critical for regulating NPC maintenance, as shown by the proximal CRE of *Mycn* and the two distal CREs of *Pbx1* (18). Consistent with this, GO enrichment analysis of the 621 genomic sites co-occupied by Eya1/Six2 revealed association with biological process of cell cycle regulation and covalent chromatin modification (Figure 2E, F). Thus, it is plausible that the activity or specificity of Eya1 in the assembly of regulatory complexes at these sites is augmented by Six2. Besides Six2, we detected general TFs, including the components of GTF2 and GTF3 complexes, RNA polymerases, and different transcriptional activators that bind to promoter sequences or enhancers (Figure 5B) and identified ∼90% of Eya1-occupied sites at proximal-promoter regions (Figure 2). Of these factors, we identified RFX1 in mass spectrometry and RFX-binding sequences at 11.9% of sites co-occupied by Eya1/Six2. RFX TFs play key functions in ciliogenesis (66), and we previously found that Rfx1 interacts with Six1 in both cultured cells and inner ear (67). Consistent with the importance of RFX in ciliogenesis, GO enrichment analysis of the Eya1/Six2-occupied sites showed association with cilium organization (Figure 2F, Supplementary Figure S2D). Thus, our analysis implicates a cooperative role between Eya1, Six2 and Rfx1 in ciliogenesis.

Beyond ensuring that promoters and enhancers are accessible to general TFs, RNA polymerases and other components of transcriptional machinery, Eya1 may also facilitate the assembly of protein complexes on REST recognition sites by interacting with chromatin remodeling factors and REST corepressors. This is supported by our RNA-seq, ChIP-seq, mass spectrometry and coIP experiments demonstrating Eya1 association to the REST recognition DNA sequences in promoter or intronic and intergenic regions of genes that were upregulated in *Eya1*cKO cells and physical association between Eya1, REST and corepressors (Figures 2, 4 and 7). We found that Rest and its corepressor Cdyl are expressed at higher levels in the NPCs and are downregulated in differentiating nephron cells (Figures 4E and 7D). Thus, we speculate that Eya1 is crucial for determining the effect of Rest on gene repression or silencing in NPCs by recruiting a corepressor complex to the REST-binding sequences. As we found that 76% of Eya1-occupied sites without H3K27ac-deposition contained REST-binding motifs, Eya1-occupancy to the REST-binding elements may be important for removing the H3K27ac mark from these sites to permit gene repression or silencing, both links tightly to chromatin status and chromatin modification in use. In the absence of Eya1, differentiation of NPCs occurs with loss of Rest repressor complex from its binding site, thus permitting upregulation or activation of a subset of nephron differentiation or disease-associated genes in *Eya1*-deficient NPCs.

Overall, our studies identify that Eya1 performs a diverse array of functions ranging from cell cycle, DNA replication and repair to transcriptional activation and REST- and PRC1-dependent repression or silencing. As Eya1 interacts with different families of chromatin remodeling complexes, one common thread linking these different functions could be the establishment of chromatin regions that are nucleosome-free so that other factors with dedicated activities can access their binding sites. Another is the existence of numerous partners that are implicated in different Eya1 functions. Similarly, Eya1 may rely on its partner proteins to mediate posttranscriptional and translational regulation. A third layer is the role of Eya1 in posttranslational modification of partner proteins. Future work to elucidate the interdependence between the multiple functions of Eya1 will contribute to further understanding of the role of the Eya1-regulatory network in maintaining the nephron progenitor state during kidney formation.

Finally, it should be noted that Eya1 occupies both REST elements and promoter sequences of the same genetic loci, including *Pax2* and *Lin28*. Thus, Eya1 may regulate the balance of their expression levels to maintain the lifespan of NPCs to ensure that a sufficient number of nephrons are produced during development. While future work is needed to reveal how Eya1’s activities are related to tissue and cell type as well as genetic context, our findings provide insight into the underlying molecular mechanisms by which increased Eya1 activity leads to various types of cancer and their metastasis.

## DATA AVAILABILITY

The RNA-seq and ChIP-seq data reported in this paper were deposited to the Gene Expression Omnibus (GEO) (GSE202957, token: ivwxumiwdbixnur). Mass Spectrometry data were deposited to ProteomeXchange (identifier PXD033955 and 10.6019/PXD033955). Protocols and materials are available upon request.

## Supplementary Material

gkac760_Supplemental_Files

## Notes

Present address: Jun Li, Chengdu Institute of Biology, Chinese Academy of Sciences, Chengdu, Sichuan 610041, China.
