# Supplementary material for: The transcriptional coactivator Eya1 exerts transcriptional repressive activity by interacting with REST corepressors and REST-binding sequences to maintain nephron progenitor identity
Source: Nucleic Acids Res. 2022 Sep 21;50(18):10343–59. doi: 10.1093/nar/gkac760 (PMC9561260; doi:10.1093/nar/gkac760)
Supplement: gkac760_Supplemental_Files [file gkac760_supplemental_files.zip › Rev-Supplementary file for Eya1 kidney paper-updated08042022.pdf]

Supplementary legends for Figure S1-S7 and Table S1-S5

Figure S1. Analysis of differentially expressed genes identified by RNA-seq. (A) Heatmap of *K*means clustering showing all differentially expressed genes between control and *Eya1*$^{cKO/cKO}$ for each biological replicate and GO enrichment analysis for each cluster. Samples "1" and "2" are biological replicates, with each RNA-seq library prepared from different 5000-cell populations FACS-sorted simultaneously. Blue or red represents down- or up-regulated differentially expressed genes. (B) GO molecular function pathway enrichment analysis for differentially expressed genes. Blue or red indicates down- or up-regulated genes.

Figure S2. Genome-wide occupancy by Eya1 in E13.5 kidneys. (A) GREAT analysis showing association of Eya1-enriched regions with terms in GO database for molecular function. (B) KEGG pathway enrichment analysis of 240 Eya1-targeted DEGs. (C) Pie charts (Galaxy toolkits) showing genomic distribution of 621 sites co-occupied by Eya1/Six2. UTR, untranslated region. (D) GREAT analysis showing association of 621 Eya1/Six2-enriched regions with terms in GO database for cellular component.

Figure S3. Genomic browser visualization of overlapping occupancy of H3K27ac, Eya1, Six2 in E13.5 kidneys and E16.5 kidneys. Six2E16.5 is from the public database (GSE39837). The panel on the right is higher magnification of the region outlined by the red box that indicates a conserved region ~60-kb upstream of the *Pax2* promoter containing a REST-binding motif occupied by Eya1. The direction of transcription is indicated by the arrow beginning at the transcription start site.

Figure S4. Eya1 occupies REST recognition sequences without H3k27ac-deposition. (A) Genomic browser visualization of overlapping occupancy of H3K27ac, Eya1 and Six2. Right panels are higher magnification of boxed area at *Podxl* and *Slc13a3* loci. (B) Genomic browser visualization showing co-occupancy of Eya1/Six2 to the promoter region of the *Rest* gene. (C) Co-immunostaining on E17.5 kidney sections showing higher levels of Rest expression in $Six2^+$ cap mesenchyme (CM). Scale bar: 100 μm.

Figure S5. Purification of Eya1 and its interacting proteins. (A,B) Search for known Eya1 interaction networks using inBio Discover™ (A) or String (B) tool showing known Eya1-interacting proteins. (C) An interactive plot showing relationship between enriched pathways for biological process enriched for proteins involved in RNA processing, posttranscriptional and translational regulation (listed on Supplementary Table S3). (D) Co-immunostaining for Hltf (red) and Six2 (green) on E16.5 kidney section. Abb.: CM, cap mesenchyme; UB, ureteric bud. Scale bar: 50 μm.

Figure S6. Analysis of ubiquitin ligase, protease, kinase and phosphatase copurified by Eya1. (A) List of E3 ubiquitin ligase, protease 26S, kinase and phosphatase copurified by Eya1. (B) A hierarchical clustering tree summarizing the correlation among significant pathways identified by GO enrichment analysis for biological process of all proteins listed in A. Pathways with many shared genes are clustered together. Bigger dots indicate more significant *P*-values.

Figure S7. REST interaction networks revealed using inBio Discover™. (A) Proteins that interact with Eya1 are indicated by green circles. (B) Co-immunostaining for Cdyl (red) and Six2

(green) on E17.5 kidney sections. Abb.: CM, cap mesenchyme; UB, ureteric bud. Scale bar: 50 μm.

Supplementary Table S1. 1181 DEGs identified by RNA-seq analysis.

Supplementary Table S2. 6022 peaks identified by Eya1 ChIP-seq analysis.

Supplementary Table S3. Posttranscriptional and translational factors copurified by Eya1.

Supplementary Table S4. Importins and exportins copurified by Eya1.

Supplementary Table S5. List of primers used for RT-qPCR.

A

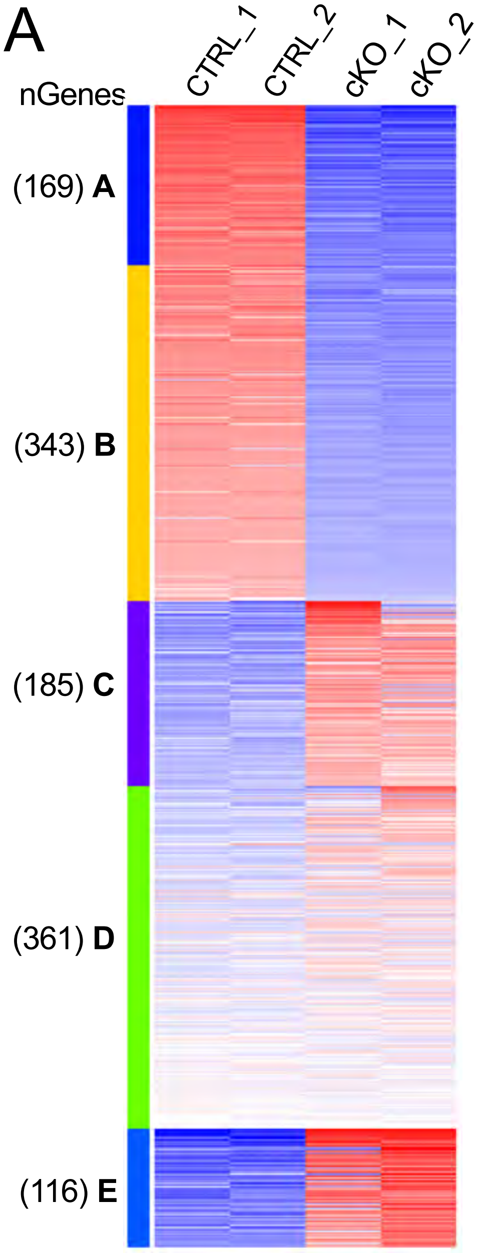

*K*means GO Biological Process

Cluster	Pathways	Adj. *P* value
A	Ureteric bud development	4.22E-08
	Kidney morphogenesis	3.04E-07
	Mesonephric tubule morphogenesis	3.04E-07
B	Nucleic acid metabolic process	2.24E-16
	Chromosome organization	1.73E-15
	Regulation of nucleobase-containing compound metabolic process	4.13E-15
	Regulation of gene expression	7.86E-13
	DNA metabolic process	1.75E-11
	Regulation of RNA metabolic process	4.58E-11
C	Renal filtration cell differentiation	0.000146915
	Glomerulus development	0.000180907
	Cell differentiation involved in kidney development	0.000387738
	Nephron development	0.001722108
	Nervous system development	0.001758525
D	System development	4.51E-23
	Movement of cell or subcellular component	1.80E-21
	Cell migration	5.87E-21
	Cell adhesion	6.76E-19
	Enzyme linked receptor protein signaling pathway	1.02E-17
	Extracellular matrix organization	6.72E-17
E	Autonomic nervous system development	1.35E-08
	Enteric nervous system development	2.31E-08
	Noradrenergic neuron differentiation	9.24E-07
	Sympathetic nervous system development	4.35E-06
	Cardiac chamber formation	1.85E-05
	Cellular hormone metabolic process	9.68E-05

B

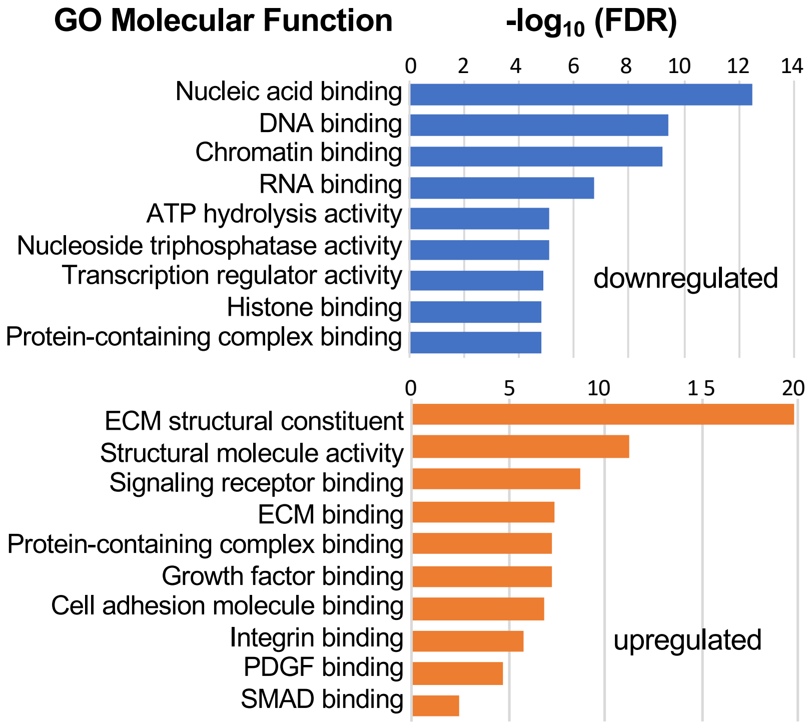

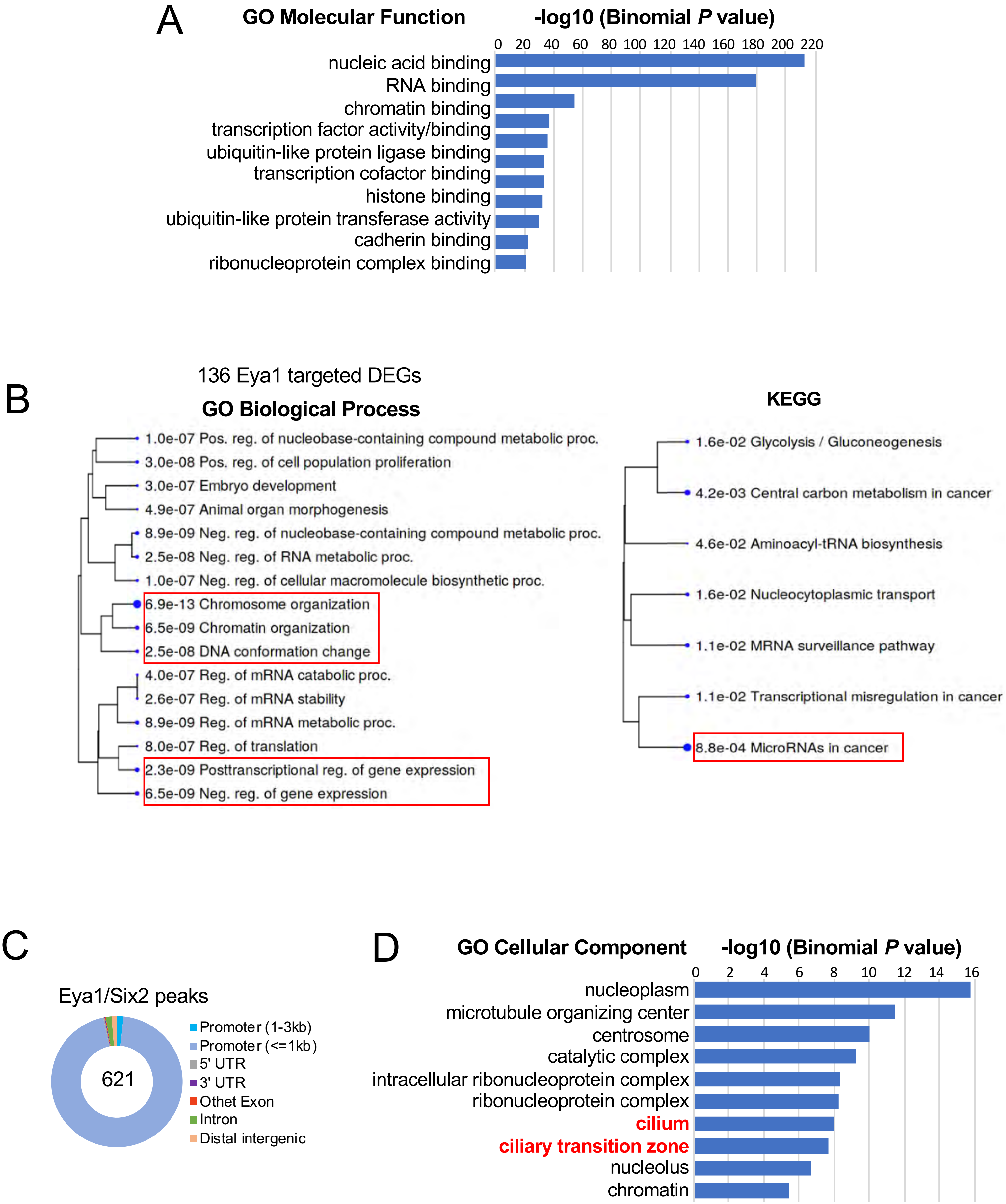
A
GO Molecular Function
-log10 (Binomial P value)
0 20 40 60 80 100 120 140 160 180 200 220
nucleic acid binding
RNA binding
chromatin binding
transcription factor activity/binding
ubiquitin-like protein ligase binding
transcription cofactor binding
histone binding
ubiquitin-like protein transferase activity
cadherin binding
ribonucleoprotein complex binding
B
136 Eya1 targeted DEGs
GO Biological Process
1.0e-07 Pos. reg. of nucleobase-containing compound metabolic proc.
3.0e-08 Pos. reg. of cell population proliferation
3.0e-07 Embryo development
4.9e-07 Animal organ morphogenesis
8.9e-09 Neg. reg. of nucleobase-containing compound metabolic proc.
2.5e-08 Neg. reg. of RNA metabolic proc.
1.0e-07 Neg. reg. of cellular macromolecule biosynthetic proc.
6.9e-13 Chromosome organization
6.5e-09 Chromatin organization
2.5e-08 DNA conformation change
4.0e-07 Reg. of mRNA catabolic proc.
2.6e-07 Reg. of mRNA stability
8.9e-09 Reg. of mRNA metabolic proc.
8.0e-07 Reg. of translation
2.3e-09 Posttranscriptional reg. of gene expression
6.5e-09 Neg. reg. of gene expression
KEGG
1.6e-02 Glycolysis / Gluconeogenesis
4.2e-03 Central carbon metabolism in cancer
4.6e-02 Aminoacyl-tRNA biosynthesis
1.6e-02 Nucleocytoplasmic transport
1.1e-02 MRNA surveillance pathway
1.1e-02 Transcriptional misregulation in cancer
8.8e-04 MicroRNAs in cancer
C
Eya1/Six2 peaks
621
Promoter (1-3kb)
Promoter (<=1kb)
5' UTR
3' UTR
Othet Exon
Intron
Distal intergenic
D
GO Cellular Component
-log10 (Binomial P value)
0 2 4 6 8 10 12 14 16
nucleoplasm
microtubule organizing center
centrosome
catalytic complex
intracellular ribonucleoprotein complex
ribonucleoprotein complex
cilium
ciliary transition zone
nucleolus
chromatin

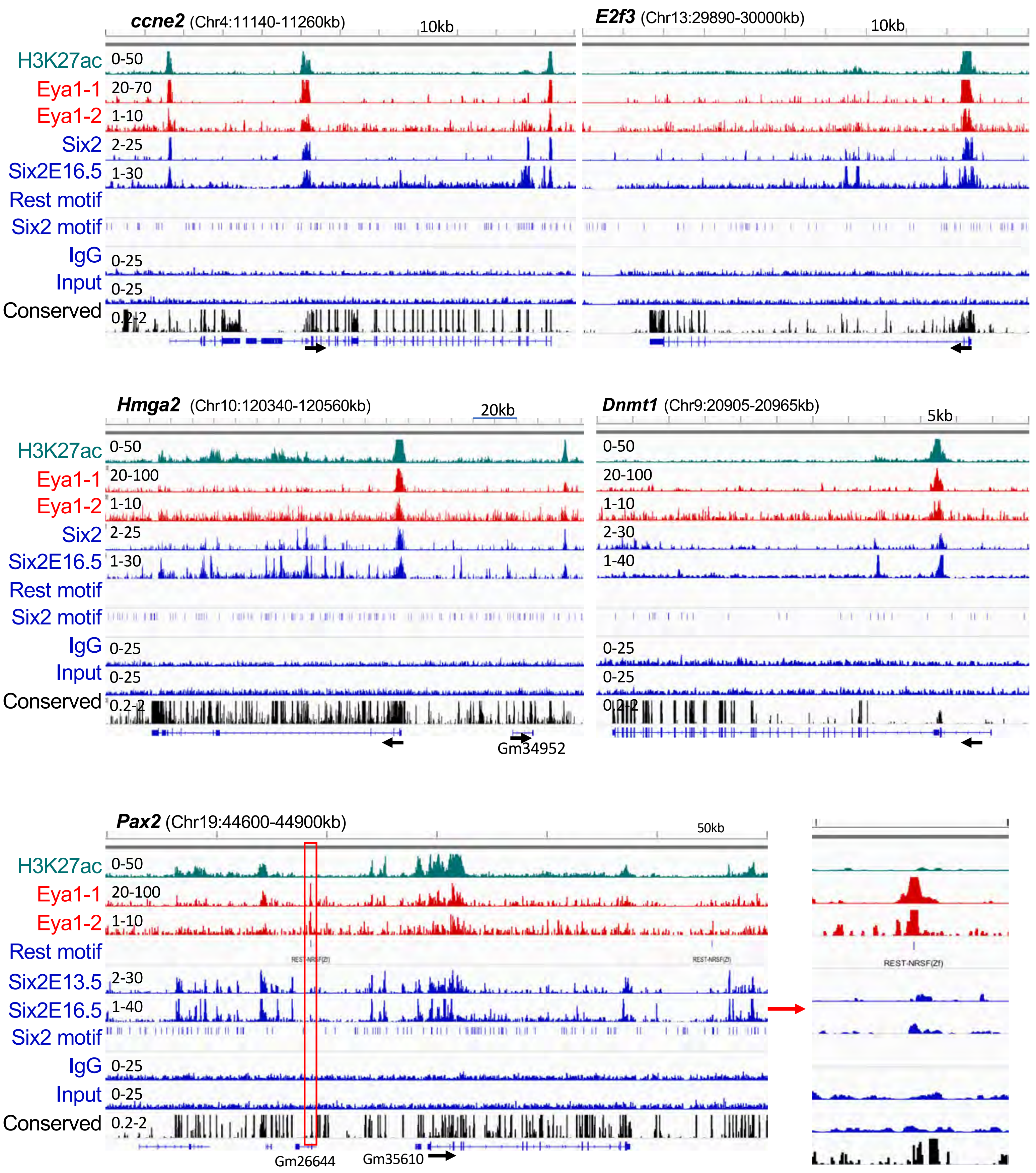
ccne2 (Chr4:11140-11260kb)
10kb
E2f3 (Chr13:29890-30000kb)
10kb
H3K27ac
Eya1-1
Eya1-2
Six2
Six2E16.5
Rest motif
Six2 motif
IgG
Input
Conserved
0-50
20-70
1-10
2-25
1-30
0-25
0-25
0.2-2
Hmga2 (Chr10:120340-120560kb)
20kb
Dnmt1 (Chr9:20905-20965kb)
5kb
20-100
2-30
1-40
Gm34952
Pax2 (Chr19:44600-44900kb)
50kb
Six2E13.5
REST-NRSF(Zf)
Gm26644
Gm35610

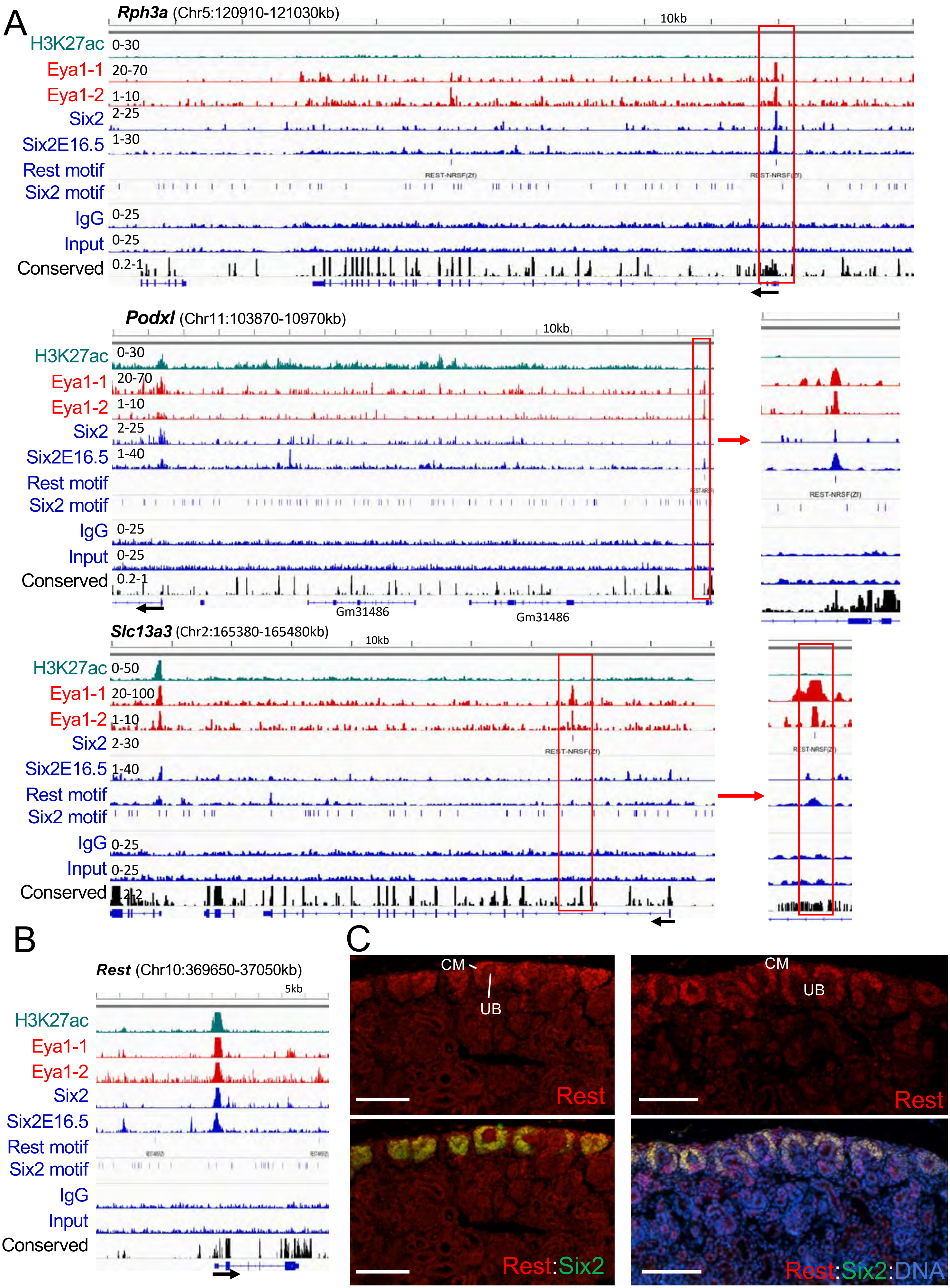

A
Rph3a (Chr5:120910-121030kb)
10kb
H3K27ac 0-30
Eya1-1 20-70
Eya1-2 1-10
Six2 2-25
Six2E16.5 1-30
Rest motif
Six2 motif
IgG 0-25
Input 0-25
Conserved 0.2-1
REST-NRSF(Zf)
Podxl (Chr11:103870-10970kb)
10kb
H3K27ac 0-30
Eya1-1 20-70
Eya1-2 1-10
Six2 2-25
Six2E16.5 1-40
Rest motif
Six2 motif
IgG 0-25
Input 0-25
Conserved 0.2-1
Gm31486
Gm31486
REST-NRSF(Zf)
Slc13a3 (Chr2:165380-165480kb)
10kb
H3K27ac 0-50
Eya1-1 20-100
Eya1-2 1-10
Six2 2-30
Six2E16.5 1-40
Rest motif
Six2 motif
IgG 0-25
Input 0-25
Conserved
REST-NRSF(Zf)
B
Rest (Chr10:369650-37050kb)
5kb
H3K27ac
Eya1-1
Eya1-2
Six2
Six2E16.5
Rest motif
Six2 motif
IgG
Input
Conserved
C
CM
UB
Rest
CM
UB
Rest
Rest:Six2
Rest:Six2:DNA

Fig. S5

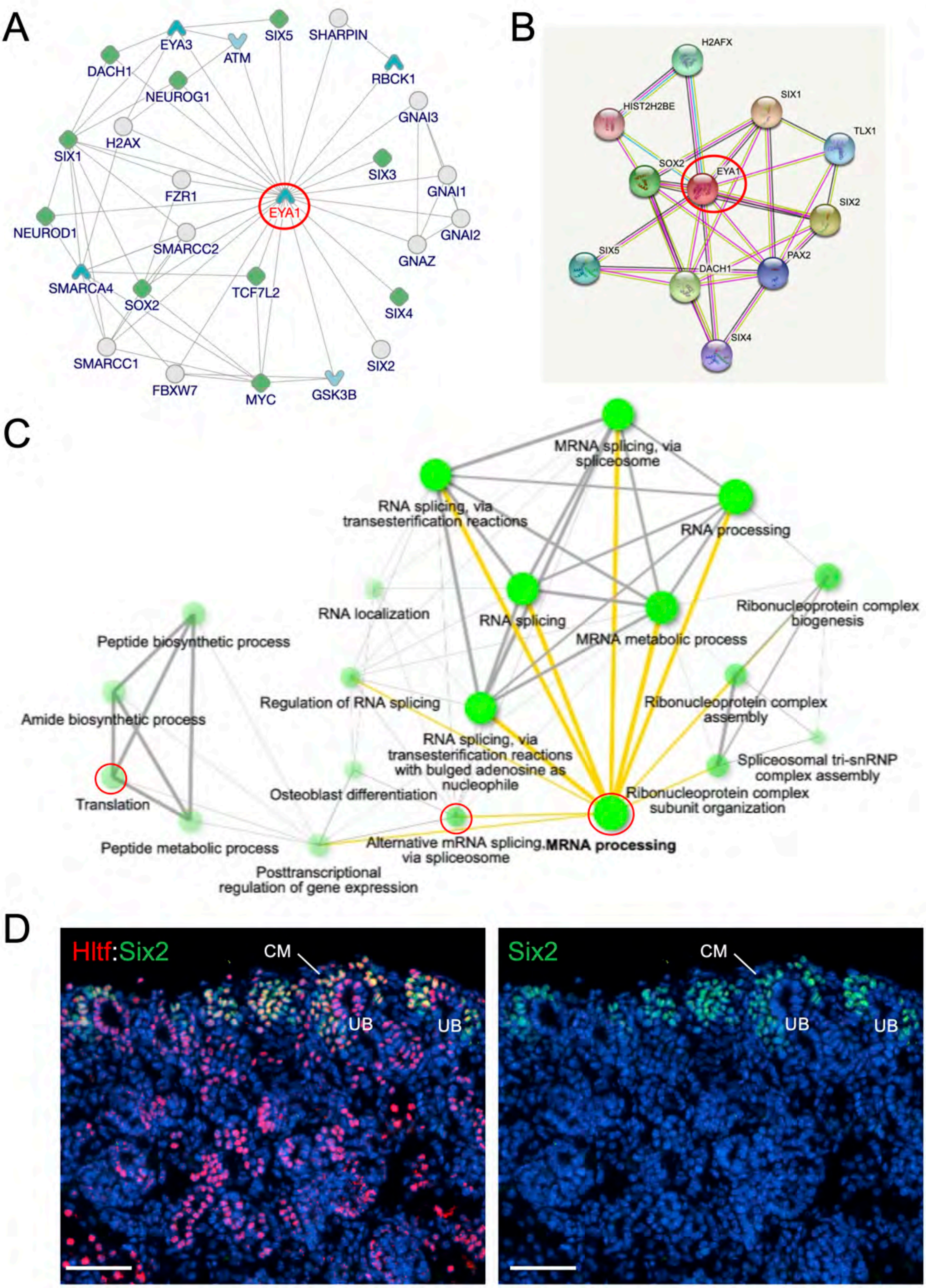
A
EYA3
ATM
SIX5
SHARPIN
DACH1
NEUROG1
RBCK1
GNAI3
H2AX
SIX1
SIX3
FZR1
GNAI1
EYA1
NEUROD1
GNAI2
SMARCC2
GNAZ
SMARCA4
TCF7L2
SOX2
SIX4
SMARCC1
SIX2
FBXW7
MYC
GSK3B
B
H2AFX
HIST2H2BE
SIX1
TLX1
SOX2
EYA1
SIX2
SIX5
DACH1
PAX2
SIX4
C
MRNA splicing, via spliceosome
RNA splicing, via transesterification reactions
RNA processing
RNA localization
RNA splicing
MRNA metabolic process
Ribonucleoprotein complex biogenesis
Peptide biosynthetic process
Regulation of RNA splicing
Ribonucleoprotein complex assembly
Amide biosynthetic process
RNA splicing, via transesterification reactions with bulged adenosine as nucleophile
Spliceosomal tri-snRNP complex assembly
Osteoblast differentiation
Ribonucleoprotein complex subunit organization
Translation
Peptide metabolic process
Alternative mRNA splicing, via spliceosome
MRNA processing
Posttranscriptional regulation of gene expression
D
Hltf:Six2
CM
UB
UB
Six2
CM
UB
UB

A

E3 ubiquitin ligase, protease 26S, kinase, and phosphatase copurified by Eya1

**Protein**	Band	Accession	Mascot[a]	Pept.[b]
TRIM33 (E3 ubiquitin-protein ligae)	B11	Q9UPN9	52	1
TRIM21	B02	P19474	75	2
RNF123 (ring finger, E3 ubiquitin-protein ligase)	B12	C9J559	55	2
UBA52	B01	P62987	21	1
	B02		44	1
	B03		73	2
	B05		49	1
	B06		68	1
	Bo7_08		168	2
	B09		119	3
	B10		29	1
	B11		66	1
	B12		78	1
PSMC2 (26S protease regulatory subunit)	B02	P35998	343	10
PSMC3	B02	P17980	169	4
PSMC4	B02	P43686	177	7
PSMC6	B01	P62333	155	4
PSMD2 (26S protease non-ATPase regulatory subunit)	B05	Q13200	101	3
PSMD3	B03	O43242	1239	19
PSMD4	B02	P55036	124	4
PSMD11	B02	O00231	37	1
MAP2K2	B01	P36507	105	2
MAP2K7	B02	O14733	156	3
MAPK7	B07_08	Q131164	113	2
PRKDC	B05	P78527	84	4
	B07_08		46	1
	B09		46	1
	B10		224	5
	B11		379	10
	B12		450	13
JAK1 (tyrosine-protein kinase)	B10	P23458	76	2
TAOK2 (serine/threonine kinase)	B12	Q9UL54	55	2
PPME1 (protein phosphatase methylesterase)	B01	Q9Y570	27	1

[a]Mascot score; [b]Number of peptide

B

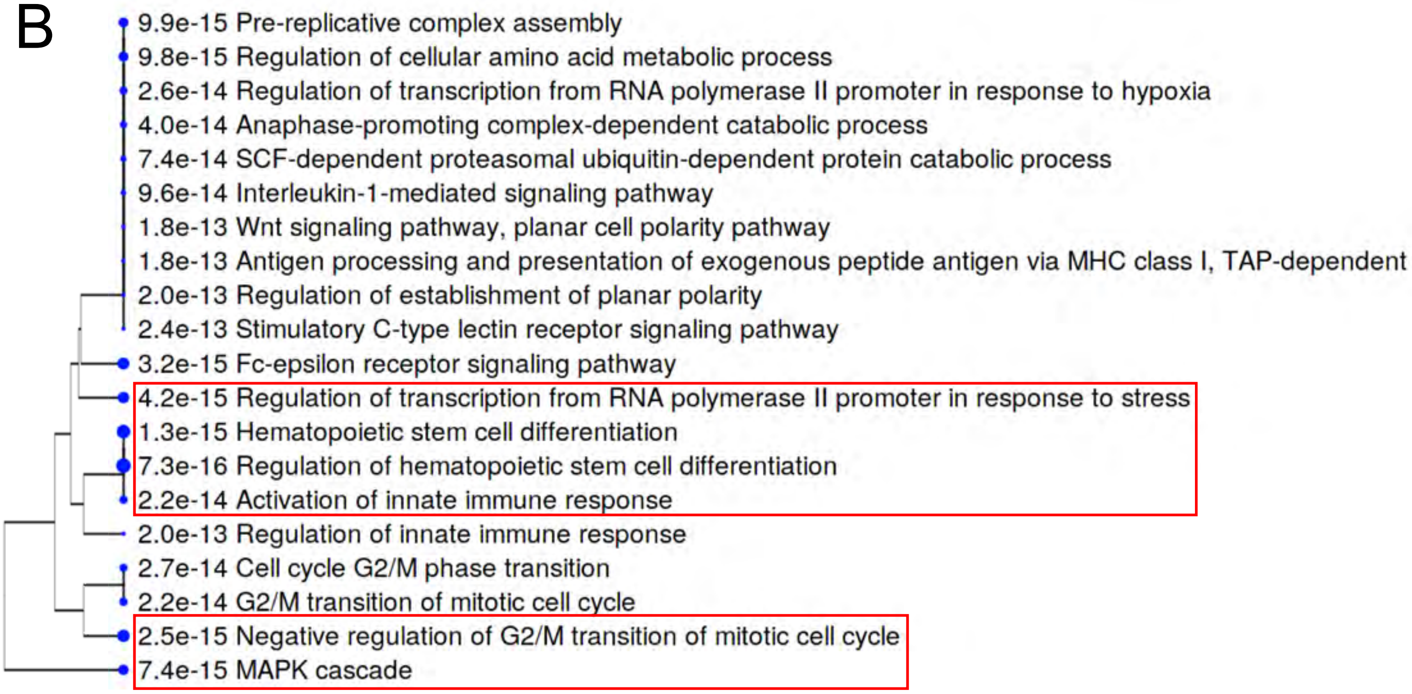

A

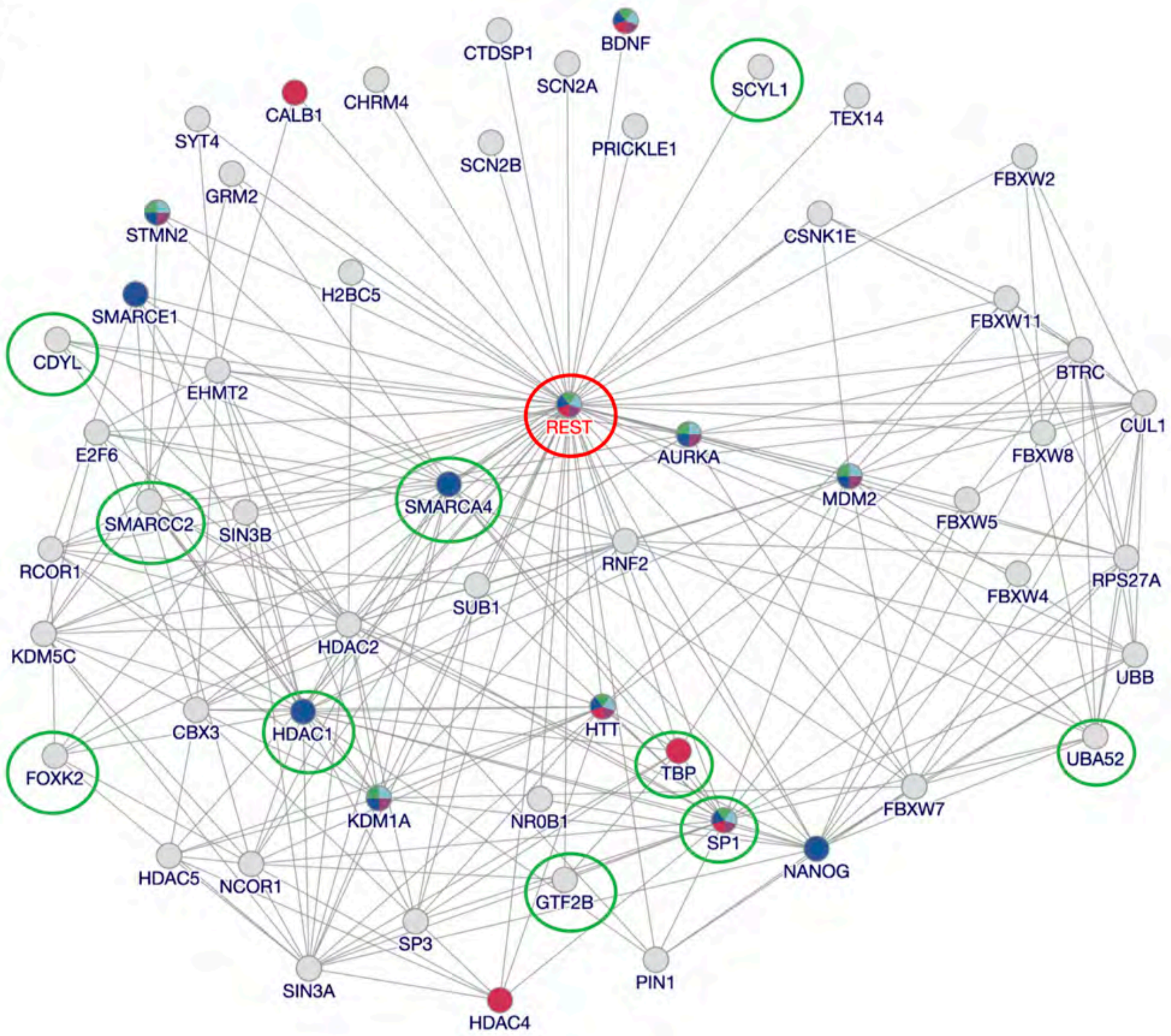
CTDSP1
BDNF
SCN2A
SCYL1
CALB1
CHRM4
TEX14
SYT4
SCN2B
PRICKLE1
GRM2
FBXW2
STMN2
CSNK1E
H2BC5
SMARCE1
FBXW11
CDYL
BTRC
EHMT2
REST
CUL1
E2F6
AURKA
FBXW8
SMARCA4
MDM2
SMARCC2
SIN3B
FBXW5
RCOR1
RNF2
RPS27A
FBXW4
SUB1
KDM5C
HDAC2
UBB
CBX3
HDAC1
HTT
TBP
UBA52
FOXK2
KDM1A
NR0B1
SP1
FBXW7
NANOG
HDAC5
NCOR1
GTF2B
SP3
SIN3A
PIN1
HDAC4

B

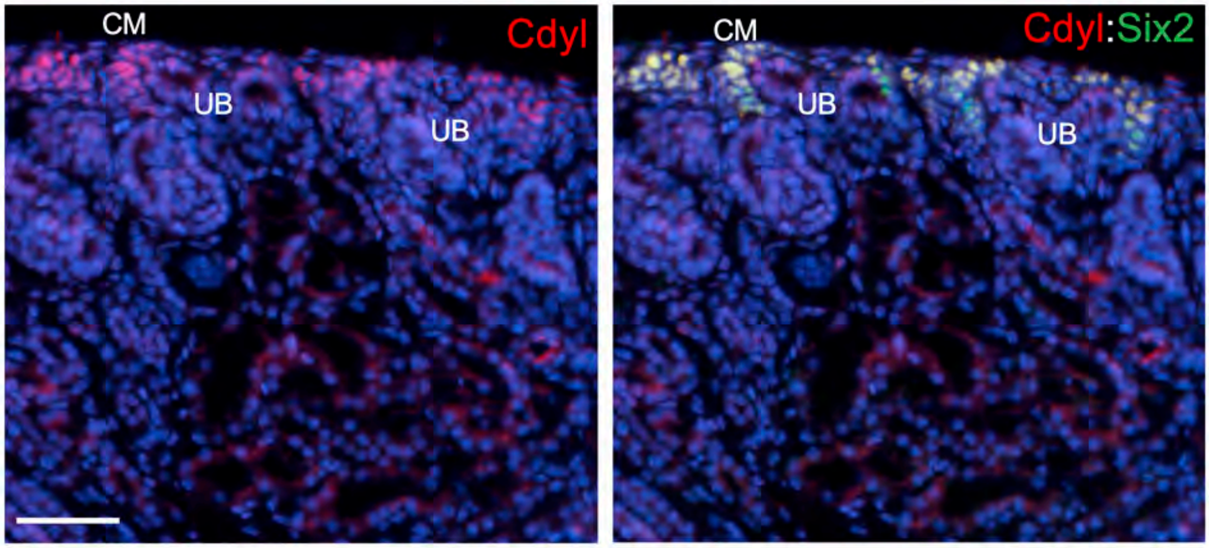
CM
Cdyl
UB
UB
CM
Cdyl:Six2
UB
UB

Supplementary Table S4. Eya1-interacting importins and exportins

Protein	Band	Accession	Mascot[a]	Pept.[b]
**Importins**				
IPO4	B09	Q8TEX9	707	14
IPO5	B09	O00410	589	13
IPO7	B09	O95373	697	18
IPO8	B02	O15397	48	1
	B09		166	5
IPO9	B09	Q96P70	918	13
**Exportins**				
XPO1	B07_08	O14980	897	14
	B09		142	5
XPOT	B06	O43592	402	10
	B06	P55060	432	7
CSE1L	B05		51	2
XPO6	B09	D3DWF9	158	5

[a]Mascot score; [b]Number of peptide

Supplementary Table S5. RT-qPCR primer sequences

Target	Forward	Reverse
Eya1a (primers F1+R2)	ATGGAAATGCAGGATCTAAC	GTGAGCTGGTCTTGGACTAA
Eya1b (primers F7+R8)	GATCTACAACACCTACAAAAA	CTATTGGAAACACAATTCCT
Six2	TGTGAGAAATGAGAGAGAGAG	CTCTACTTTCCTTTCGAGTT
Lin28a	GTGTTCTGTATTGGGAGTG	CAGCTTGCATTCCTTGGCAT
Lin28b	CAGAAACGGAAAAAGACTTAG	GAACTTCCCAGTTCCTCAGCA
Cited 1	CAGCTTCAGAAGCTTAATAGC	GAGACATCAGCACCTCCTCAT
Pax2	ACGACACCTTGTGTTTGGTC	TCGTCAGTTCCTTCCAGGCT
Col1a1	TCTACTGCAACATGGAGACA	GTCATGCTCTCTCCAAACC
Chga	CCTGAGTGCCCAACAGCAA	TTCCATCTCCATCCACTGCCT
Nphs1	GACAAGAAGACTCCAGCTGA	GTATAATGATCCCTAAATAG
Aldob	GACTTGGGAT CCTCTAACTA	CTCCAAGCATTGAAGTCACA
β-actin	CATTGTTACCAACTGGGACGA	GAAGGTCTCAAACATGATCTG
Gapdh	AGCCAAAAGGGTCATCATCTC	TGATGGCATGGACTGTGGTCA
